# Synthesis, In Vitro Anti-Inflammatory Activity, and HRMS Analysis of New Amphetamine Derivatives

**DOI:** 10.3390/molecules28010151

**Published:** 2022-12-24

**Authors:** Stanimir Manolov, Iliyan Ivanov, Dimitar Bojilov, Paraskev Nedialkov

**Affiliations:** 1 Department of Organic Chemistry, Faculty of Chemistry, University of Plovdiv, 4000 Plovdiv, Bulgaria; 2 Department of Pharmacognosy, Faculty of Pharmacy, Medical University of Sofia, 1000 Sofia, Bulgaria

**Keywords:** amphetamine, ibuprofen, flurbiprofen, ketoprofen, naproxen, carprofen, amides, HRMS, amfens, in vitro, toxicity

## Abstract

Herein, we report the obtaining of new hybrid molecules of amphetamine with different profens (amfens). The obtained amfens are characterized by their melting points, UV, ^1^H–, ^13^C–NMR, and HRMS spectra. A complete and detailed mass spectral analysis of the newly obtained derivatives of amphetamine with ibuprofen, flurbiprofen, ketoprofen, naproxen, and carprofen was performed. In vitro inhibition of albumin denaturation of each new compound was assessed, and they showed significant activity. The IC_50_ values of the obtained amphetamine-profen derivatives ranged from 92.81 to 159.87 µg/mL. This indicates that the new hybrids inherit the anti-inflammatory properties of profens. Using in silico method, the toxicity was also calculated. The obtained results are given in LD_50_ values. Depending on the route of administration, the amfens are less toxic compared to the standard amphetamine.

## 1. Introduction

For many years, scientists have been fascinated by the synthesis of amphetamine and its derivatives [1]. To date, numerous organic synthetic transformations have been reported. The constant search for similar biofunctional molecules is the driving force behind the discovery of novel approaches to the synthesis of amphetamine and its derivatives [1]. 

Amphetamine is a compound that consists of a phenyl ring connected to an amino group by a two-carbon hydrocarbon chain and a methyl group at the α-carbon. It is a psychoactive substance that induces feelings of cheerfulness, strength, and lightness in people who are severely physically exhausted. The compound and its derivatives are used to treat depression, narcolepsy, and obesity [2]. 

**Figure 1:**
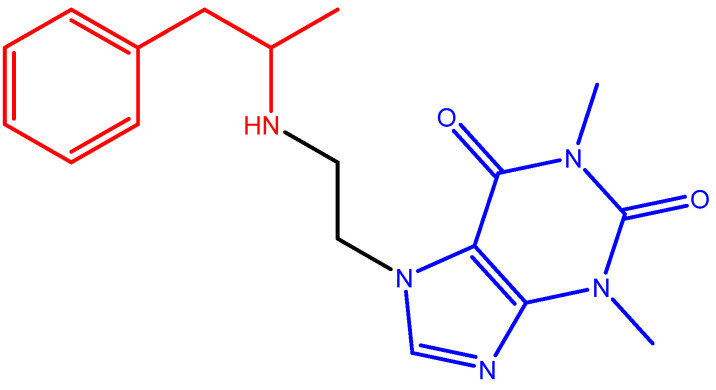
Structural formula of captagon.

Fenethylline or Captagon, is a combination of amphetamine (given in red) and theophylline (given in blue) (Figure 1) and was created in 1961, thought to be a milder form of amphetamine [3].

It creates a feeling of immense power and euphoria, while at the same time keeping you awake for a long time. The two components, amphetamine and theophylline, act separately on the brain and enhance each other’s psychoactive properties. It is used to treat narcolepsy, fatigue, and behavioral disorders such as "minimal brain dysfunction." Unlike amphetamines, captagon does not raise blood pressure, which means it can also be used to treat people with cardiovascular disease. However, it turned out to be highly addictive, and in 1986, its use was banned in most countries around the world [4].

Some of the most used amphetamine derivatives are 3,4-methylenedioxymethamphetamine (MDMA), 4-iodo-2,5-dimethoxyamphetamine (DOI), norfenfluramine, deprenyl, 4-methylthioamphetamine (MTA), bupropion, and phentermine, which are well-known for their entactogenic, hallucinogenic, anorectic, antiparkinsonian, flat liner, antidepressant, and anorectic properties, respectively [5].

Nonsteroidal anti-inflammatory drugs (NSAIDs) are used worldwide to treat pain and inflammation [6]. Profens make the most important group of nonsteroidal anti-inflammatory drugs (NSAIDs), and in many countries, some of them are freely sold. Chemically, all profens bear the characteristic 2-aryl-propionic acid moiety. They are optically active compounds due to the presence of chiral carbon (alpha-carbon of propionic acid) [7]. 

**Figure 2:**
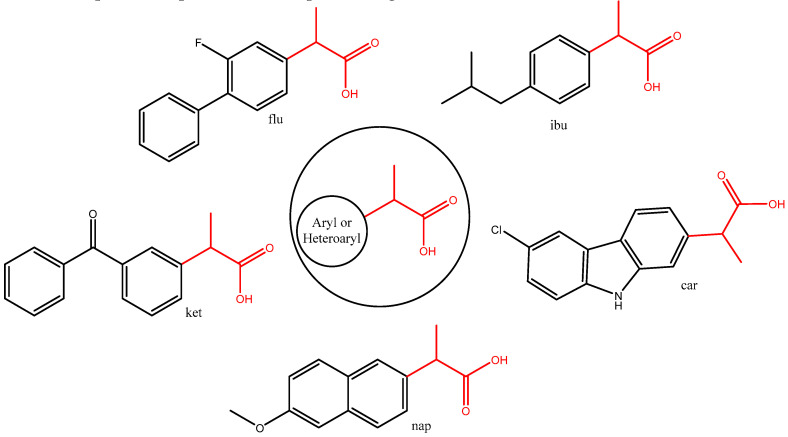
Structural formulas of Ibuprofen (ibu), flurbiprofen (flu), ketoprofen (ket), naproxen (nap), and carprofen (car).

Some of the most well-known and widely used profens are ibuprofen, flurbiprofen, ketoprofen, naproxen, and carprofen (Figure 2.).

This was the reason for obtaining hybrid molecules that structurally combine both the amphetamine and the profen skeleton. Those two classes of compounds can be easily combined via an amide bond. 

Amides represent some of the most important and valuable organic functional groups in naturally occurring molecules, pharmaceuticals, agrochemicals, and polymers. The synthesis of amides is of great importance in organic and medicinal chemistry. Improved and innovative methods for the synthesis of amides are in high demand, both from the chemical and pharmaceutical industries. Various amides are present in about 25% of the best-selling pharmaceutical products and in many other important medicinal compounds [8]. 

In this regard, we aimed to synthesize new hybrid molecules combining the powerful central nervous system stimulant actions of amphetamine with the strong analgesic properties of profens due to their anti-inflammatory effect. 

## 2. Results and Discussion

### 2.1. Synthesis

An extensive literature review revealed that amphetamine is a structure that has piqued the interest of synthetic chemists for over a century. In this regard, we were looking for compounds that contained both an amphetamine and a profen fragment. For convenience, further, in the article, we will call them amfens (amphetamine-profens) for short. 

Creating new structures that combine amphetamine and profen molecules could be useful for studying their biological activity. When the two structures are combined, a completely new molecule is formed that has the biological properties of both amphetamine and profen. This is the driving force behind our efforts to create new amfen molecules.

**Scheme 1:**
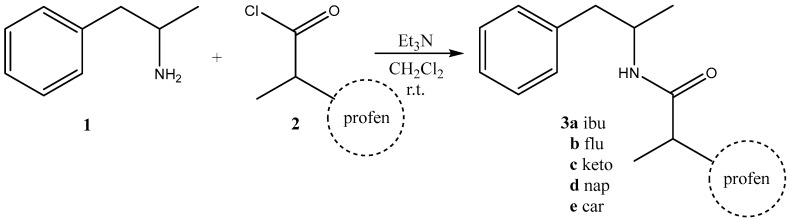
Synthesis of new amfens **3a**–**e**.

The classic method for the synthesis of amides was applied to obtain the final amfens, the Schotten–Baumann method, which consists of the acylation of amphetamine **1** with acid chlorides **2** (Scheme 1). Table 1 shows the yields of the obtained products. All further analyzes were performed on the diastereomeric mixtures. 

The reaction mixture was stirred in an electromagnetic stirrer at room temperature. Тriethylamine was used as a hydrogen chloride acceptor. The progress of the reaction is monitored by thin-layer chromatography. The reaction was complete fully and no starting amphetamine was observed. The final products were isolated and additionally purified by filtration throughout neutral Al_2_O_3_.

Examining the ^1^H–NMR (Supplementary Materials Figures S1–S5) data in detail, it can be seen that all the signals are fully consistent with the corresponding molecule. To describe the signals, the same were compared with the spectral data of the corresponding parent drugs. The characteristic doublet for 6 protons from the ibuprofen residue in compound **3a** emerges at 0.83 ppm. The NH signal comes out for each amfen in the region between 5.09 and 5.25 ppm. The signals for the methyl groups appear as doublets. The doublet for the methyl group from amphetamine is seen in all new compounds from 0.94 to 0.98 ppm, and the –CH_3_ signal from profen part is seen as a doublet at 1.38–1.47 ppm. In the region around 7 ppm, all the aromatic protons appear. ^13^C-NMR spectra (Supplementary Materials Figures S6–S10) of the obtained compounds also confirmed the structure of the compounds. The UV spectra of all compounds are also available in the supplementary file (Figures S11–S15). The complete description of the structures from the NMR data was also demonstrated with the molecular ion of each compound. The performed mass spectral analysis unequivocally confirmed both the structure and the purity of the obtained amfens (Supplementary Materials Figures S16–S20). Further fragmentation allowed us to study their structures and decay pathways in detail. 

### 2.2. Mass Analysis

**Scheme 2:**
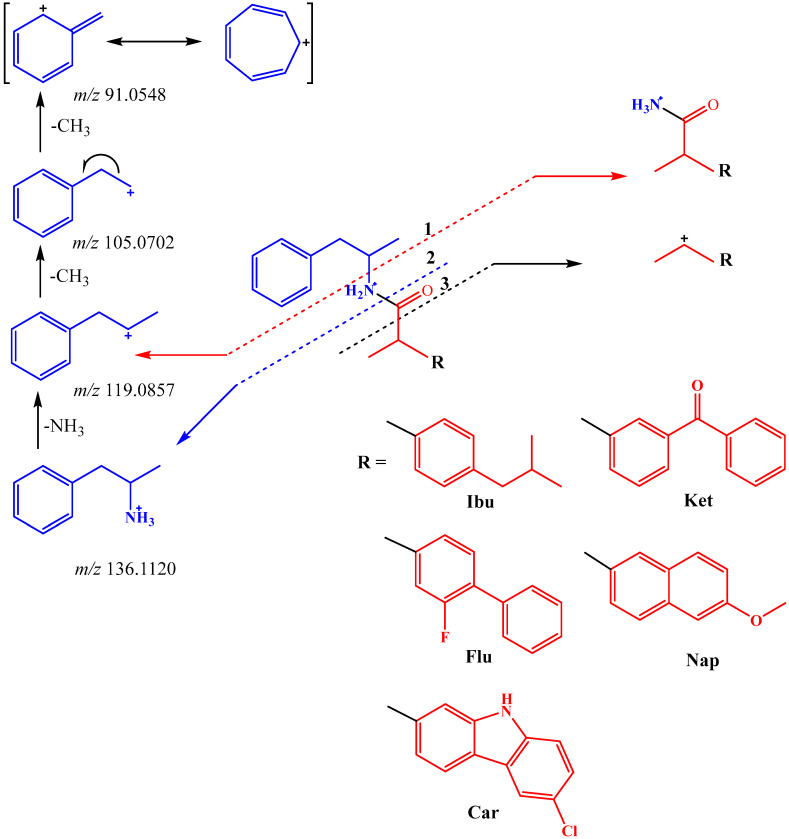
Possible fragmentation pathways of amphetamine profens **3a**–**e** under ESI-MS/MS conditions. Ibu-ibuprofen, Ket-ketoprofen, Flu-flurbiprofen, Nap-naproxen, Car-carprofen.

We investigated the fragmentation of newly synthesized amfens with structurally different profens in this study. The profen residue distinguishes the newly synthesized compounds **3a**–**e**. We used mass spectrometry to investigate the structure of the new amphetamine hybrid molecules. ESI was used in positive ionization mode. The fragmentation of amphetamine derivatives **3a**–**e** follows a mechanism depicted in Scheme 2.

The structural fragment that connects amphetamine to the various profens is -CH-NH-C(O)-C(CH_3_)-. Several fragmentation pathways originate from here. The main fragmentation pathways of compounds **3a**–**e** are associated with the cleavage of C-N (path 1 (red)), N-C (path 2 (blue)), and C-C (path 3) bonds.

Pathway 2 is associated with cleavage of the N-C bond. It is mainly characteristic of amphetamine fragmentation. The resulting amphetamine ion of *m/z* 136 undergoes loss of a neutral NH_3_ molecule, resulting in an ion of *m/z* 119. The subsequent loss of – CH_3_ yields an ion of *m/z* 91, which is resonance stabilized to a tropylium cation. The ions with *m/z* 136, 119, and 91 are amphetamine-specific ions (Scheme 2).

Paths 1 and 3 are characteristic only for the fragmentation of the amfens. Pathway 1 is related to the cleavage of the C-N bond, resulting in an amide cation of the corresponding profen (*m/z* 206 for ibuprofen, *m/z* 244 for flurbiprofen, *m/z* 254 for ketoprofen, *m/z* 230 for naproxen, *m/z* 273 for carprofen) (Supplementary Materials Figures S16–S20).

**Scheme 3:**
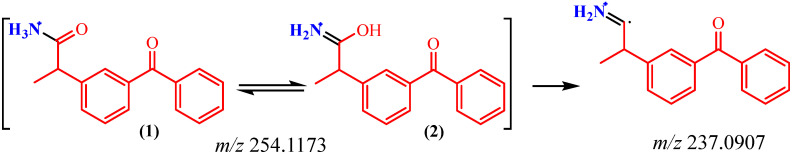
Proposed scheme of fragmentation of resonance-stabilized ketoprofen amide. Tautomeric form (2) undergoes loss of OH group, yielding radical ion m/z 237.

**Scheme 4:**
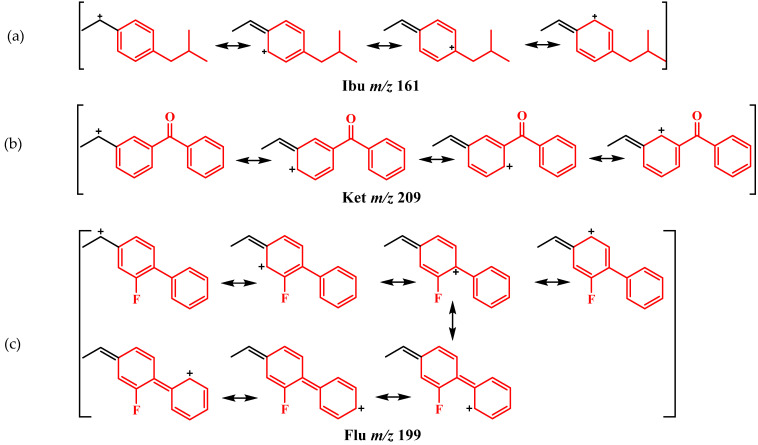
Resonance-stabilized profen cations with non-condensed aromatic cores. (**a**) Ibu-ibuprofen, (**b**) Ket-ketoprofen and (**c**) Flu-flurbiprofen.

**Scheme 5:**
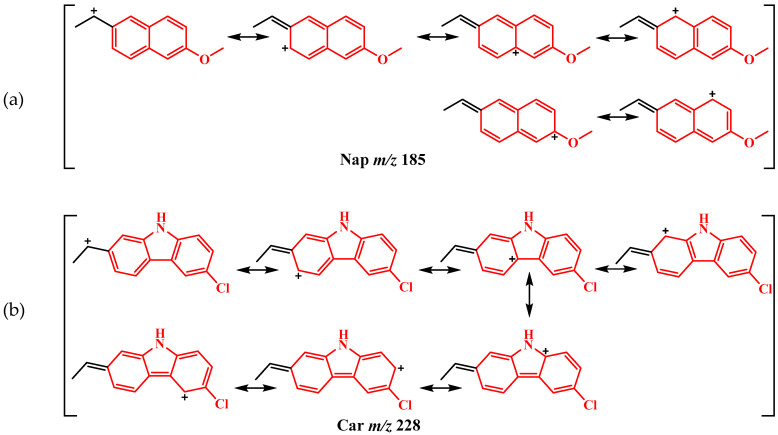
Resonance-stabilized profen cations with condensed aromatic cores. (**a**) Nap-naproxen, (**b**) Car-carprofen.

The mass spectrum shows that the resulting ions have low intensity. The profenamide cation is stable and does not undergo further fragmentation except for ketoprofen. The fragmentation of the ketoprofen amide cation (*m/z* 254) is probably due to the fact that the tautomeric form (2) undergoes loss of an OH group, and a radical ion with *m/z* 237 is obtained (Scheme 3):

Path 3 C-C bond cleavage yields stable aromatic cations with *m/z* 161 for ibuprofen, *m/z* 199 for flurbiprofen, *m/z* 209 for ketoprofen, *m/z* 185 for naproxen, *m/z* 228 for carprofen (Scheme 4a–c, Scheme 5a,b, Supplementary Materials Figures S13–S16).

Their stability is due to the formation of resonance-stable cations (Scheme 4a–c, Scheme 5a,b). The ion intensity increases as the aromatic rings in the profen structure increase. The profens with condensed cores, naproxen and carprofen, have the highest intensity. 

### 2.3. Anti-Inflammatory Activity

Amfens are hybrid molecules that are made up of fragments with different properties and structures. Amphetamine is characterized by its psychotropic properties. It inhibits the enzyme monoamine oxidase (EC 1.4.3.4), a flavoenzyme that catalyzes the oxidation of biogenic amines, increasing the levels of the neurotransmitters norepinephrine, serotonin, and dopamine in the brain [9,10]. Profens are used as NSAIDs. They have the property of inhibiting COX1 and COX2, which oxidize arachidonic acid to prostaglandins [6].

Until now, no information has been found in the literature about the synthesis of hybrid molecules, which in their structures contain fragments with psychotropic and anti-inflammatory properties. Applying such an approach can lead to the appearance of two effects: one is to increase or slow down the psychotropic effect and the second is that the resulting amfens exhibit good anti-inflammatory properties. What application they will find depends mostly on what properties they will inherit.

In the present work, we investigated the in vitro anti-inflammatory activity of the newly synthesized compounds **3a**–**e**, evaluated as inhibition of albumin denaturation (IAD).

Inflammation is the response of living tissues to injury. It involves a complex array of enzyme activation, mediator release, fluid extravasation, cell migration, tissue breakdown, and repair [11]. The denaturation of proteins is a well-documented cause of inflammation in rheumatoid arthritis [12]. The obtained amfens were screened for the inhibition of albumin denaturation. This method provides information as to which albumin is protected from denaturation when heated. For this purpose, we have used human albumin. 

The profens ibuprofen, flurbiprofen, ketoprofen, naproxen, and carprofen as structural fragments in amfens have proven anti-inflammatory properties, we used them as comparative standards.

**Figure 3:**
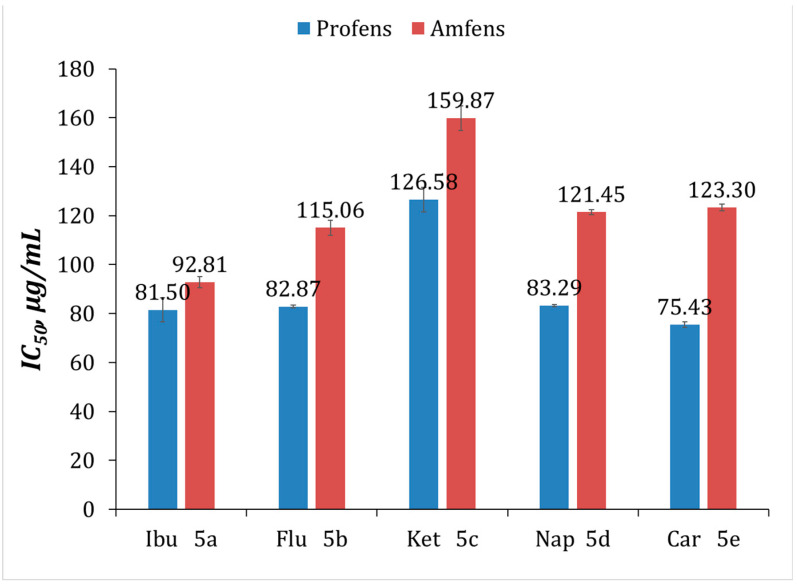
Inhibition of albumin denaturation (IAD) of profens and amfens **3a**–**e**. As standards, anti-inflammatory medications, such as ibuprofen (Ibu), flulbiprofen (Flu), ketoprofen (Ket), naproxen (Nap), and carprofen (Car), were utilized. The results are presented as IC_50_ (µg/mL).

The percentages of inhibition of synthesized amfens are presented in Figure 3. The results of the study are presented as IC_50_. The IC_50_ values of profens estimated as IAD range from 75.43 to 126.58 µg/mL (Figure 3). The IC_50_ values of the resulting amfens ranged from 92.81 to 159.87 µg/mL. Carprofen exhibits the highest anti-inflammatory activity. We assume that this is due to the fact that it contains 9*H*-Carbazole in its structure. Amfens showed anti-inflammatory activity, although they resulted less potent than their analogous free profens. As the number of aromatic nuclei increases, the anti-inflammatory activity decreases, i.e., the lipophilic character of the newly synthesized amfens (**3a**–**c**) is enhanced. This trend is not observed in amfens with condensed aromatic cores. Perhaps, therefore, amfens do not reproduce the same order of anti-inflammatory activity as profens. Overall, the results of the in vitro studies indicate that the amfens exhibit significant activity. We derive important information from the experiment, which confirms that the resulting amfens possess the property of protecting the albumin molecule from denaturation upon heating. 

The purpose of the experiment is to find out what properties the new hybrid molecules (amfens) will inherit. The experiment carried out by IAD proves that amfens inherit the properties of profens.

### 2.4. Prediction of Acute Rat Toxicity by Gusar Software

The toxicity of amphetamines is a widely discussed topic. Amphetamines produce both peripheral and central sympathomimetic effects. Their lethal complications include severe hyperthermia and hypertension, dysrhythmias, ischemia, dissections, and intracranial hemorrhage [13]. In order to analyze the toxicity of the newly obtained amphetamine derivatives, we used GUSAR software (Plovdiv, Bulgaria).

Gusar software was created to make QSAR/QSPR models on the basis of the appropriate training sets. The software offers in silico prediction of LD_50_ values for rats with four types of administration, oral, intravenous, intraperitoneal, and subcutaneous [14].

We used the software for quantitative in silico toxicity prediction of LD_50_ values of the obtained amfens **3a**–**e**. Table 2 presents the obtained results as LD_50_ values in mg/kg. 

**Figure 4:**
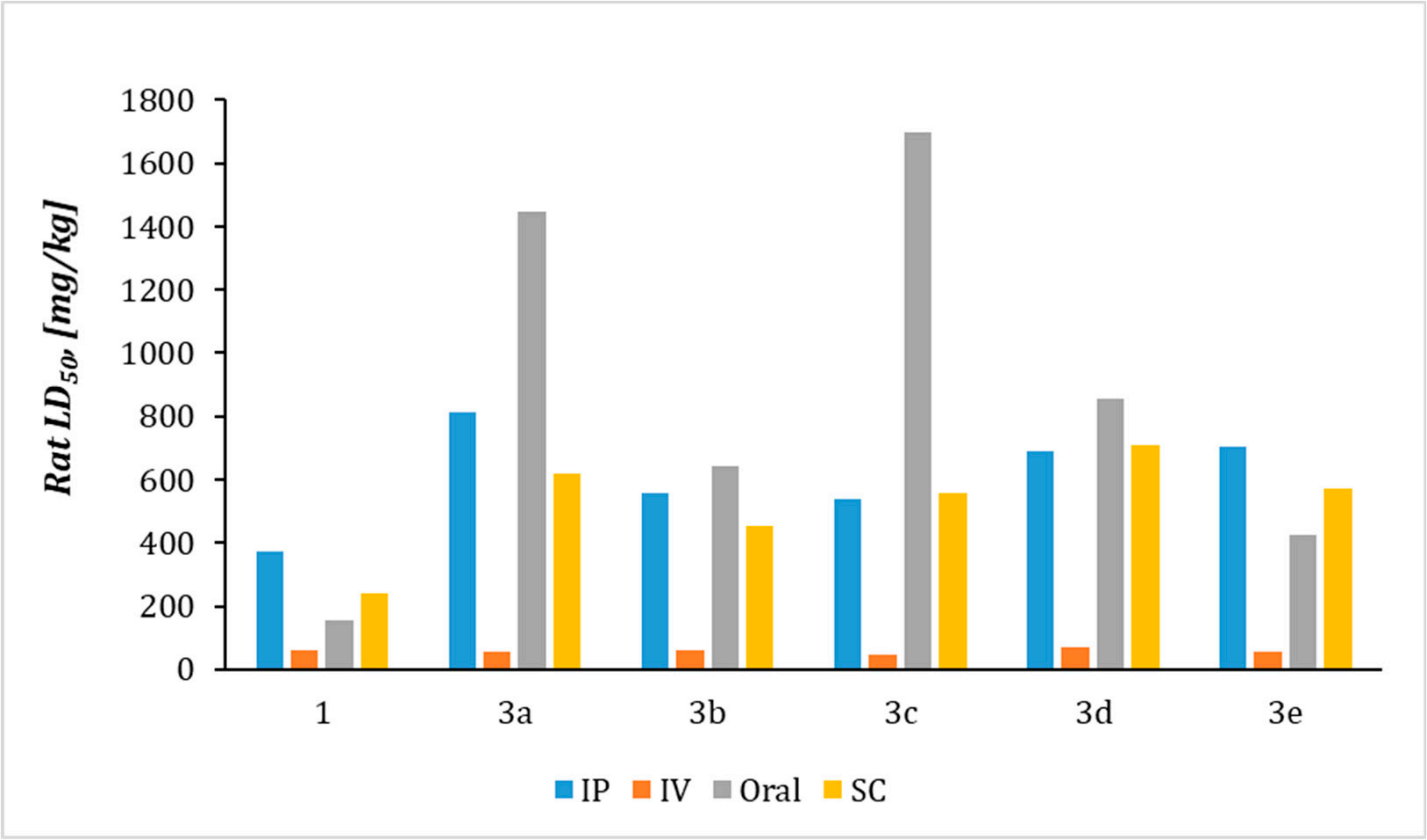
Acute rat toxicity predicted by GUSAR.

Observing the obtained data (Table 2), it can be seen that all amfens **3a**–**e** show lower calculated toxicity compared to amphetamine **1**, on the three types of administration: Intraperitoneal, subcutaneous, and oral. Amfens **3a**–**e** seem to be with similar calculated toxicity compared to the standard amphetamine **1** when administrated intravenous (IV). This can be attributed to the fact that the intravenous drug administration provides the most complete drug availability with a minimum delay. For amfens **3a** and **3b,** the following order of decreasing calculated toxicity was observed depending on the route of administration: IV > SC > IP > Oral, while for amfens **3c** and **3d,** the order is as follows: IV > IP > SC > Oral (Figure 4). Only with compound **3e**, as well as with the standard amphetamine **1**, the calculated toxicity changes in the order: IV > Oral > SC > IP. The results of this study show that the oral administration of this family of drugs is more beneficial, reducing the toxic effect. Amfens **3a** and **3c** were seen to dramatically reduce the toxic effect when being orally administrated by 9- to 11- fold compared to amphetamine **1** (Figure 4).

## 3. Materials and Methods

### 3.1. Synthesis

The reagents were purchased from commercial suppliers (Sigma-Aldrich S.A. and Riedel-de Haën, Sofia, Bulgaria) and used as received. A Bruker Avance Neo 400 was used for the recording of NMR spectral data (BAS-IOCCP—Sofia, Bruker, Billerica, MA, USA). All compounds were analyzed in CDCl_3_ at 400 and 101 MHz for ^1^H-NMR and ^13^C-NMR, respectively. Chemical shifts were determined to tetramethylsilane (TMS) (δ = 0.00 ppm) as an internal standard. The coupling constants are given in Hz. Recorded NMR spectra were taken at room temperature (approx. 295 K). The MS analysis was carried out on a Q Exactive Plus high-resolution mass spectrometer (HRMS) with a heated electrospray ionization source (HESI-II) (Medical University of Sofia, Thermo Fisher Scientific, Inc., Bremen, Germany) equipped with a Dionex Ultimate 3000RSLC ultrahigh-performance liquid chromatography (UHPLC) system (Thermo Fisher Scientific, Inc., Bremen, Germany). For the TLC analysis, precoated 0.2 mm Fluka silica gel 60 plates (Merck KGaA, Darmstadt, Germany) were used. 

Corresponding acyl chlorides **2a**–**e** (1 mmol) were added to a solution of amphetamine **1** (1 mmol) in dichloromethane (15 mL) at room temperature. Triethylamine (1.2 mmol) was added after 10 min. After 30 min, the reaction mixture was washed with diluted hydrochloric acid (H_2_O:HCl = 4:1 *v/v*), a saturated solution of Na_2_CO_3_, and water. The organic layer was dried using anhydrous Na_2_SO_4_, concentrated, and filtered on a short column with neutral Al_2_O_3_.

*2-(4-isobutylphenyl)-N-(1-phenylpropan-2-yl)propanamide***3a**.

^1^H NMR (400 MHz, CDCl_3_) δ 7.19 – 7.08 (m, 4H, Ar), 7.06 – 7.00 (m, 4H, Ar), 6.98 – 6.93 (m, 2H, Ar), 5.09 (d, *J* = 8.3 Hz, 1H, NH), 4.14 (tdq, *J* = 13.1, 8.1, 6.5 Hz, 1H, CH(NH)(CH_3_)(CH_2_)), 3.37 (dq, *J* = 9.7, 7.2 Hz, 1H, CH(CH_3_)), 2.68 – 2.52 (m, 2H, CH_2_), 2.39 (dd, *J* = 7.2, 4.5 Hz, 2H, CH_2_), 1.79 (dh, *J* = 13.5, 6.8, 6.3 Hz, 1H, CH(CH_3_)_2_), 1.38 (dd, *J* = 10.7, 7.2 Hz, 3H, CH_3_), 0.94 (dd, *J* = 12.0, 6.7 Hz, 3H, CH_3_), 0.83 (dd, *J* = 6.6, 4.1 Hz, 6H, 2xCH_3_). ^13^C NMR (101 MHz, CDCl_3_) δ 173.8(C=O), 140.6(Ar), 138.3(Ar), 137.9(Ar), 129.6(Ar), 129.4(Ar), 128.3(Ar), 127.4(Ar), 126.4(Ar), 46.9(CH), 46.1(CH_2_CHNH), 45.0(CH_2_), 42.3(COCHCH_3_), 30.2(CH(CH_3_)_2_), 22.4(CH(CH_3_)_2_), 19.9(NHCHCH_3_), 18.4(CH(CH_3_)). UV λ_max_, MeOH: 234 (ε = 21000) nm, 285 (ε = 990) nm. HRMS Electrospray ionization (ESI) m/z calcd for [M+H]^+^ C_22_H_30_NO^+^ = 324.2322, found 324.2316 (mass error Δ_m_ = −1.85 ppm).

*2-(2-fluoro-[1,1’-biphenyl]-4-yl)-N-(1-phenylpropan-2-yl)propanamide***3b** [15].

*2-(3-benzoylphenyl)-N-(1-phenylpropan-2-yl)propanamide***3c**.

^1^H NMR (400 MHz, CDCl_3_) δ 7.70 (dddd, *J* = 8.4, 3.3, 2.4, 1.9 Hz, 2H), 7.63 – 7.58 (m, 2H), 7.54 – 7.49 (m, 1H), 7.44 – 7.33 (m, 4H), 7.19 – 7.05 (m, 3H), 7.02 – 6.97 (m, 1H), 6.90 – 6.84 (m, 1H), 5.21 (s, 1H), 4.16 (tdq, *J* = 13.2, 8.3, 6.6 Hz, 1H), 3.45 (qd, *J* = 7.1, 4.7 Hz, 1H), 2.71 – 2.59 (m, 1H), 2.58 (d, *J* = 8.4 Hz, 1H), 1.41 (dd, *J* = 7.2, 1.7 Hz, 3H), 0.98 (dd, *J* = 14.7, 6.7 Hz, 3H). ^13^C NMR (101 MHz, CDCl_3_) δ 196.55((Ar_2_)C=O), 172.58(CHNHC=O), 141.89(Ar), 138.01(Ar), 137.50(Ar), 137.45(Ar), 132.59(Ar), 131.51(Ar), 130.05(Ar), 129.37(Ar), 129.35(Ar), 129.35(Ar), 129.05(Ar), 128.39(Ar), 128.36(Ar), 126.47(Ar), 47.07(CHNH), 45.97(CH_2_), 42.10(CHCH_3_), 19.94(CH_3_CHNH), 18.50(CH_3_CHNH). UV λ_max_, MeOH: 233 (ε = 29,000) nm, 278 (ε = 5900) nm. HRMS Electrospray ionization (ESI) m/z calcd for [M+H]^+^ C_25_H_26_NO_2_^+^ = 372.1958, found 372.1951 (mass error Δ_m_ = −1.88 ppm).

*2-(6-methoxynaphthalen-2-yl)-N-(1-phenylpropan-2-yl)propanamide***3d**.

^1^H NMR (400 MHz, CDCl_3_) δ 7.66 – 7.58 (m, 2H), 7.54 – 7.46 (m, 1H), 7.25 – 7.16 (m, 1H), 7.12 – 6.97 (m, 4H), 6.94 – 6.86 (m, 2H), 6.78 – 6.71 (m, 1H), 5.10 (t, *J* = 7.9 Hz, 1H), 4.15 (ddp, *J* = 19.6, 8.2, 6.5 Hz, 1H), 3.85 (d, *J* = 3.3 Hz, 3H), 3.53 (dq, *J* = 11.7, 7.2 Hz, 1H), 2.66 – 2.52 (m, 1H), 2.52 (dd, *J* = 6.2, 1.7 Hz, 1H), 1.47 (dd, *J* = 11.6, 7.2 Hz, 3H), 0.93 (dd, *J* = 15.0, 6.7 Hz, 3H). ^13^C NMR (101 MHz, CDCl_3_) δ 173.61(C=O), 157.77((Ar)C-O-CH3), 137.75(Ar), 137.44(Ar), 136.62(Ar), 136.30(Ar), 133.76(Ar), 129.40(Ar), 129.02(Ar), 128.15(Ar), 127.60(Ar), 126.25(Ar), 126.10(Ar), 119.13(Ar), 105.66(Ar), 55.36(-OCH_3_), 47.12(CHNH), 45.71(CH_2_CHNH), 42.01(CHCH_3_), 19.88(NHCHCH_3_), 18.28(CHCH_3_). UV λ_max_, MeOH: 253 (ε = 17,700) nm, 283 (ε = 5200) nm, 351 (ε = 1800) nm. HRMS Electrospray ionization (ESI) m/z calcd for [M+H]^+^ C_23_H_26_NO_2_^+^ = 348.1958, found 348.1951 (mass error Δ_m_ = −2.01 ppm).

*2-(6-chloro-9H-carbazol-2-yl)-N-(1-phenylpropan-2-yl)propanamide***3e**.

^1^H NMR (400 MHz, CDCl_3_) δ 8.83 (s, 1H), 7.92 (dq, *J* = 8.0, 1.0 Hz, 1H), 7.84 (dd, *J* = 8.0, 3.0 Hz, 1H), 7.29 – 7.19 (m, 3H), 7.08 – 6.88 (m, 5H), 6.77 – 6.70 (m, 1H), 5.25 (dd, *J* = 14.3, 8.2 Hz, 1H), 4.18 (ddq, *J* = 17.0, 8.2, 6.5 Hz, 1H), 3.55 (dq, *J* = 10.8, 7.1 Hz, 1H), 2.66 – 2.46 (m, 2H), 1.47 (dd, *J* = 7.2, 6.5 Hz, 3H), 0.96 (dd, *J* = 22.6, 6.6 Hz, 3H). ^13^C NMR (101 MHz, CDCl_3_) δ 173.73(C=O), 140.76(Ar), 139.92(Ar), 139.62(Ar), 138.29(Ar), 137.36(Ar), 129.32(Ar), 128.33(Ar), 128.16(Ar), 126.24(Ar), 125.86(Ar), 124.81(Ar), 120.79(Ar), 119.90(Ar), 119.56(Ar), 111.80(Ar), 109.63(Ar), 47.70, 45.97, 42.04(CHCH_3_), 20.06(NHCHCH_3_), 18.64(CHCH_3_). UV λ_max_, MeOH: 260 (ε = 32,000) nm, 283 (ε = 17,000) nm, 321 (ε = 16,600) nm. HRMS Electrospray ionization (ESI) m/z calcd for [M+H]^+^ C_24_H_24_ClN_2_O^+^ = 391.1572, found 391.1563 (mass error Δ_m_ = −2.30 ppm).

### 3.2. In Vitro Analysis

#### Inhibition of Albumin Denaturation (IAD)

In vitro analysis of anti-inflammatory activity was assessed as inhibition of albumin denaturation (IAD). The analysis was performed according to Sakat method [16] with minor modifications [17]. The experiment was performed with human albumin. The solution of albumin (1%) was prepared in distilled water (pH 7.4). The tested compounds/standard were dissolved firstly in 1.2 mL DMF and PBS up to 25 ml, so the final concentration of the stock solution is 1000 μg/mL. Then, a series of working solutions with different concentrations (20–500 μg/mL) in PBS was prepared. The reaction mixture contained 2 mL test sample/standard of different concentrations and 1 mL albumin (1 %). The mixture was incubated at 37 ^o^C for 15 min and then heated at 70 ^o^C for 15 min in water bath. After cooling, the turbidity was measured at 660 nm with a spectrophotometer (Camspec M508, Leeds, England). The experiment was performed three times. Percentage inhibition of albumin denaturation (IAD) was calculated against control. The control sample is albumin with the same concentration dissolved in distilled water.

(1) $$\%\mathrm{IAD}=\left[\frac{A_{\mathrm{control}}{-A}_{\mathrm{sample}}}{A_{\mathrm{control}}}\right]*100$$

## 4. Conclusions

In conclusion, we have successfully obtained four new hybrid molecules containing an amphetamine skeleton and profen part. The newly obtained amfens are fully characterized. A detailed mass spectral analysis of the newly obtained amfens was performed. In vitro analysis showed that the amfens inherit the anti-inflammatory properties of profens. Compounds **3a**–**e** are expected to be less toxic, especially via oral administration, based on the calculated values via GUSAR software. The obtained predicted toxicity results for oral administration of the newly synthesized amfens show much lower toxicity values in comparison with the amphetamine. This makes them promising and interesting for future biological evaluation.

## Figures and Tables

**Table 1:** Yields and melting points of amfens **3**.

Compound 3	Yield, %	mp, ^o^C
**a**	94	46–49
**b**	90	112–115
**c**	90	Oil
**d**	95	112–114
**e**	85	126–127

**Table 2:** Acute rat toxicity predicted via GUSAR software.

Compound	Rat LD_50_ [mg/kg]			
	IP	IV	Oral	SC
**1**	370.7^4a^	60.25^4a^	153.4^3b^	238.5^4a^
**3a**	813.6^5a^	56.99^4a^	1448^4a^	617.3^4a^
**3b**	557.9^5a^	59.79^4a^	641.8^4a^	454.6^4a^
**3c**	539.6^5a^	47.6^4a^	1696^4a^	555.7^4a^
**3d**	691.7^5a^	70.67^4a^	856.5^4a^	706.9^4a^
**3e**	702.1^5a^	54.6^4a^	426^4a^	572.5^4a^

IP-intraperitoneal, IV-intravenous, SC-subcutaneous, Applicability domain: a-compound falls in the applicability domain of models; b-compound does not fall in the applicability domain of models. The superscript numbers show the acute rodent toxicity classification by OECD (The Organization for Economic Co-operation and Development). OECD classifies the chemicals into five categories, where 1 is highly toxic and 5 is practically non-toxic.

## Data Availability

The data presented in this study are available in this article and in the supporting Supplementary Material.

## Supplementary Materials

The following supporting information can be downloaded at: https://www.mdpi.com/article/10.3390/molecules28010151/s1; Figure S1: ^1^H-NMR spectrum of compound **3a**; Figure S2: ^1^H-NMR spectrum of compound **3b**; Figure S3: ^1^H-NMR spectrum of compound **3c**; Figure S4: ^1^H-NMR spectrum of compound **3d**; Figure S5: ^1^H-NMR spectrum of compound **3e**; Figure S6: ^13^C-NMR spectrum of compound **3a**; Figure S7: ^13^C-NMR spectrum of compound **3b**; Figure S8: ^13^C-NMR spectrum of compound **3c**; Figure S9: ^13^C-NMR spectrum of compound **3d**; Figure S10: ^13^C-NMR spectrum of compound **3e**, Figure S11: UV spectrum of compound **3a**; Figure S12: UV spectrum of compound **3b**; Figure S13: UV spectrum of compound **3c**; Figure S14: UV spectrum of compound **3d**; Figure S15: UV spectrum of compound **3e**; Figure S16: Mass spectrum of **3a** obtained by positive ion ESI-MS/MS; Figure S17: Mass spectrum of **3b** obtained by positive ion ESI-MS/MS; Figure S18: Mass spectrum of **3c** obtained by positive ion ESI-MS/MS; Figure S19: Mass spectrum of **3d** obtained by positive ion ESI-MS/MS; Figure S20: Mass spectrum of **3e** obtained by positive ion ESI-MS/MS.

## Sample Availability

Samples of compounds **3a**–**e** are available from the authors.
