# Supplementary material for: Synthesis, In Vitro Anti-Inflammatory Activity, and HRMS Analysis of New Amphetamine Derivatives
Source: Molecules. 2022 Dec 24;28(1):151. doi: 10.3390/molecules28010151 (PMC9822421; doi:10.3390/molecules28010151)
Supplement: Supplementary file 1 [file molecules-28-00151-s001.zip › molecules-2090531-supplementary.pdf]

Supplementary Materials:

Synthesis, in vitro anti-inflammatory activity, and HRMS analysis of new amphetamine derivatives

Stanimir Manolov, Iliyan Ivanov, Dimitar Bojilov, Paraskev Nedialkov

All supplementary materials for compound **3b** are previously published [15].

Table of Contents:

Figure S1. ^{1}H-NMR spectrum of compound **3a** page **3**

Figure S2 ^{1}H-NMR spectrum of compound **3b** page **4**

Figure S3. ^{1}H-NMR spectrum of compound **3c** page **5**

Figure S4. ^{1}H-NMR spectrum of compound **3d** page **6**

Figure S5. ^{1}H-NMR spectrum of compound **3e** page **7**

Figure S6. ^{13}C-NMR spectrum of compound **3a** page **8**

Figure S7. ^{13}C-NMR spectrum of compound **3b** page **9**

Figure S8. ^{13}C-NMR spectrum of compound **3c** page **10**

Figure S9. ^{13}C-NMR spectrum of compound **3d** page **11**

Figure S10. ^{13}C-NMR spectrum of compound **3e** page **12**

Figure S11. UV spectrum of compound **3a** page **13**

Figure S12. UV spectrum of compound **3b** page **14**

Figure S13. UV spectrum of compound **3c** page **15**

Figure S14. UV spectrum of compound **3d** page **16**

Figure S15. UV spectrum of compound **3e** page **17**

Figure S16. Mass spectrum of **3a** obtained by positive ion ESI-MS/MS page **18**

Figure S17. Mass spectrum of **3b** obtained by positive ion ESI-MS/MS page **19**

Figure S18. Mass spectrum of **3c** obtained by positive ion ESI-MS/MS page **20**

Figure S19. Mass spectrum of **3d** obtained by positive ion ESI-MS/MS page **21**

Figure S20. Mass spectrum of **3e** obtained by positive ion ESI-MS/MS page **22**

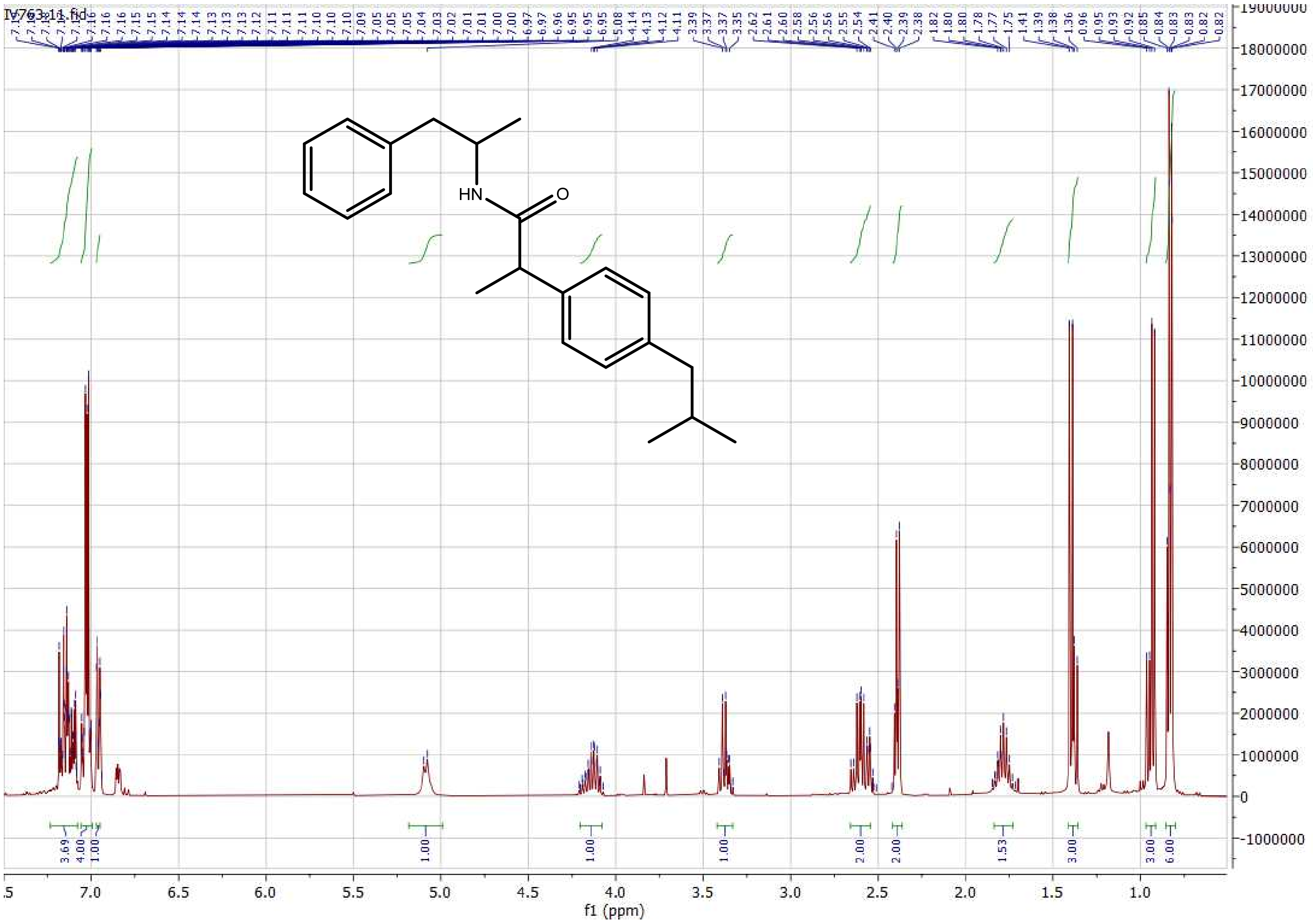

Figure S1. ^{1}H-NMR spectrum of compound **3a**.

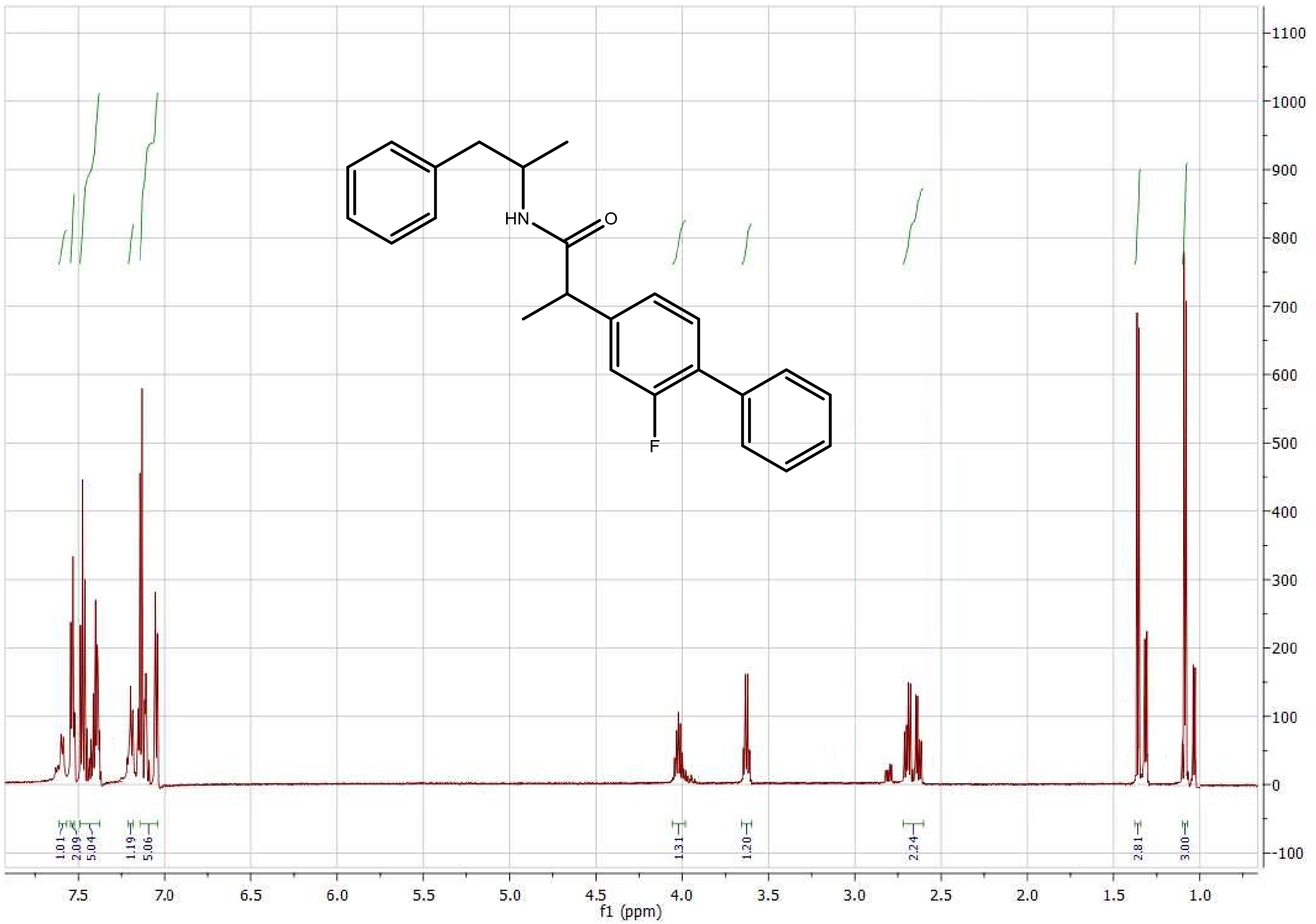

Figure S2. ^{1}H-NMR spectrum of compound **3b**.

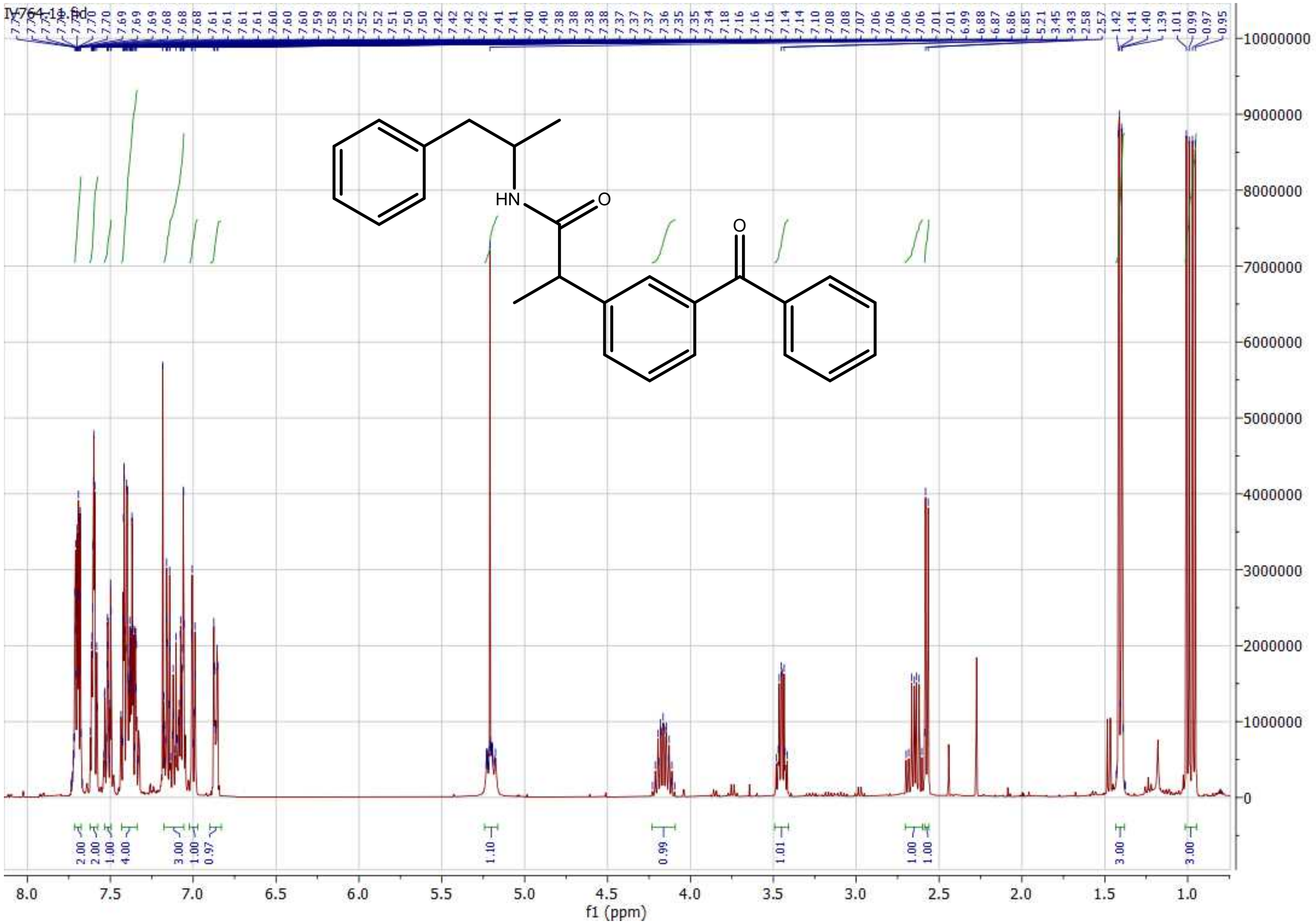

Figure S3. ^{1}H-NMR spectrum of compound **3c**.

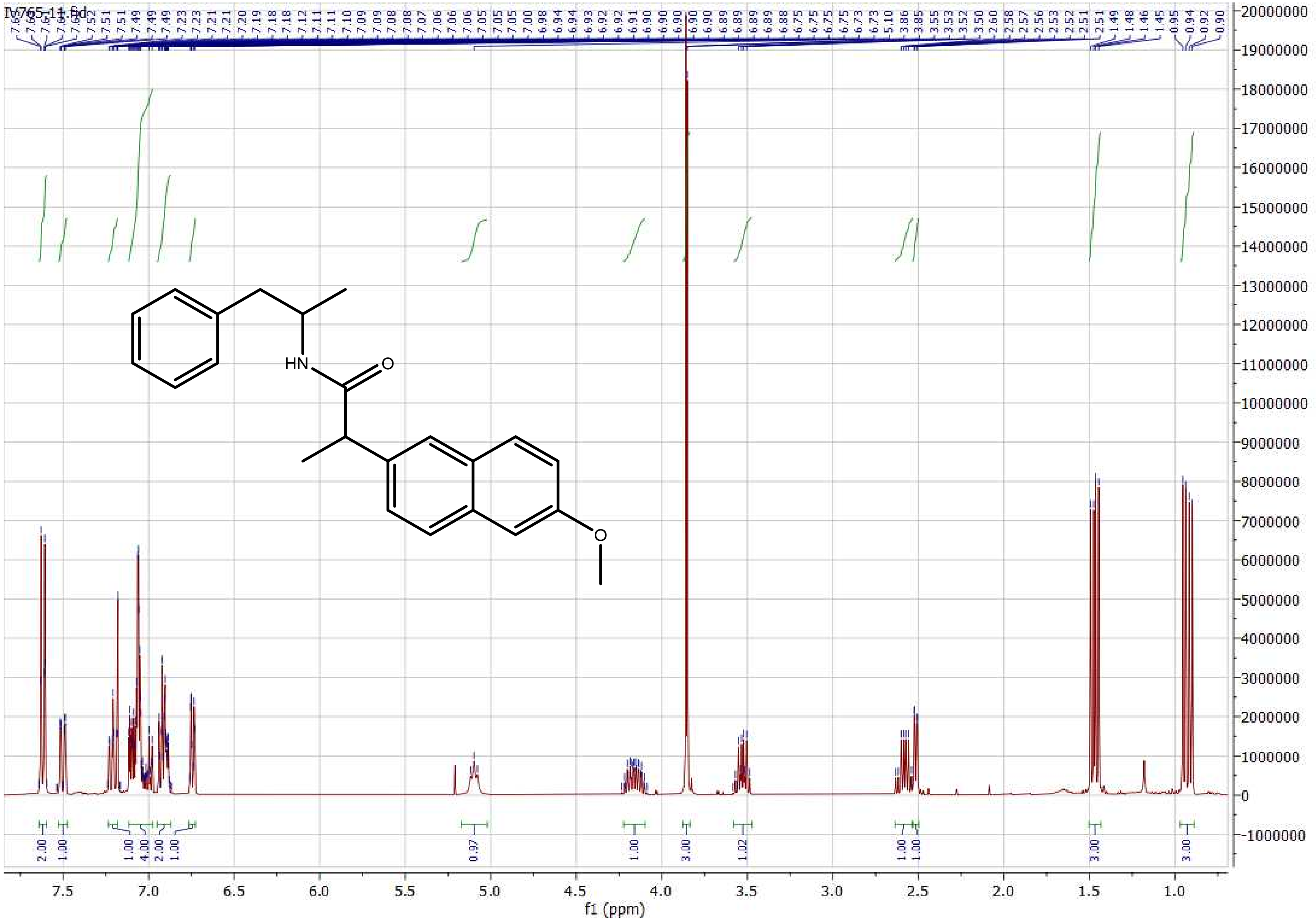

Figure S4. ^{1}H-NMR spectrum of compound **3d**.

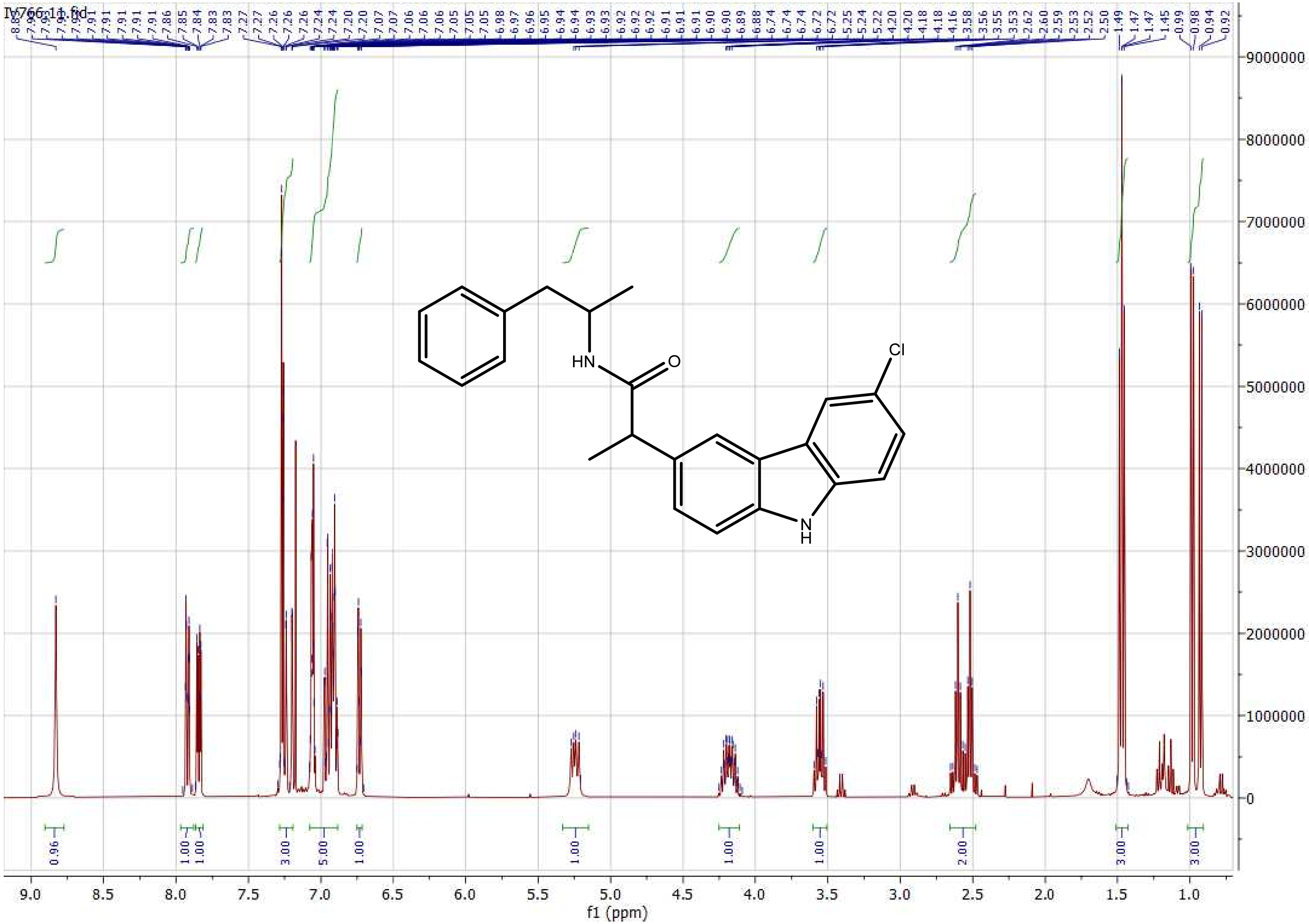

Figure S5. ^{1}H-NMR spectrum of compound **3e**.

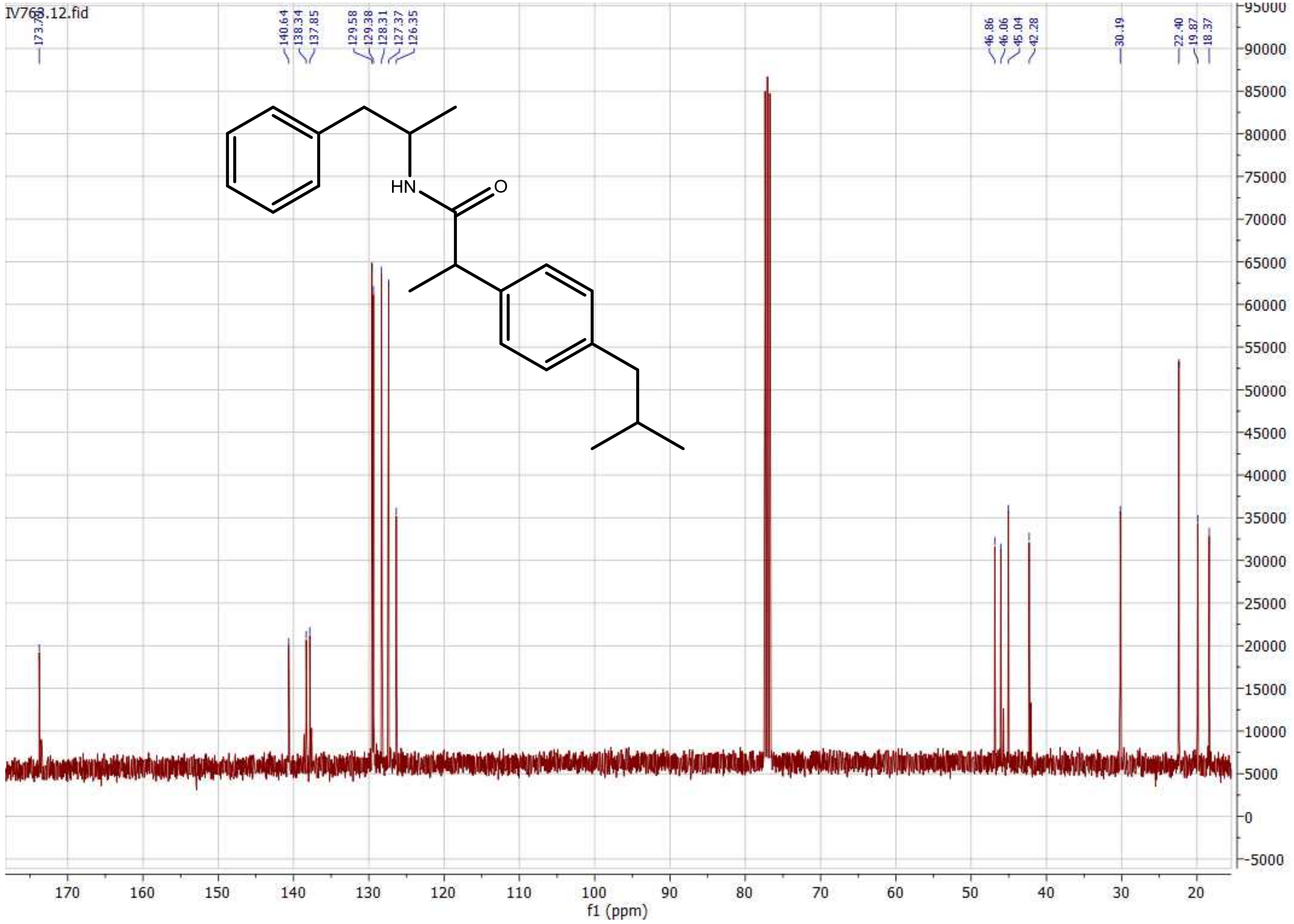

Figure S6. ^{13}C-NMR spectrum of compound **3a**.

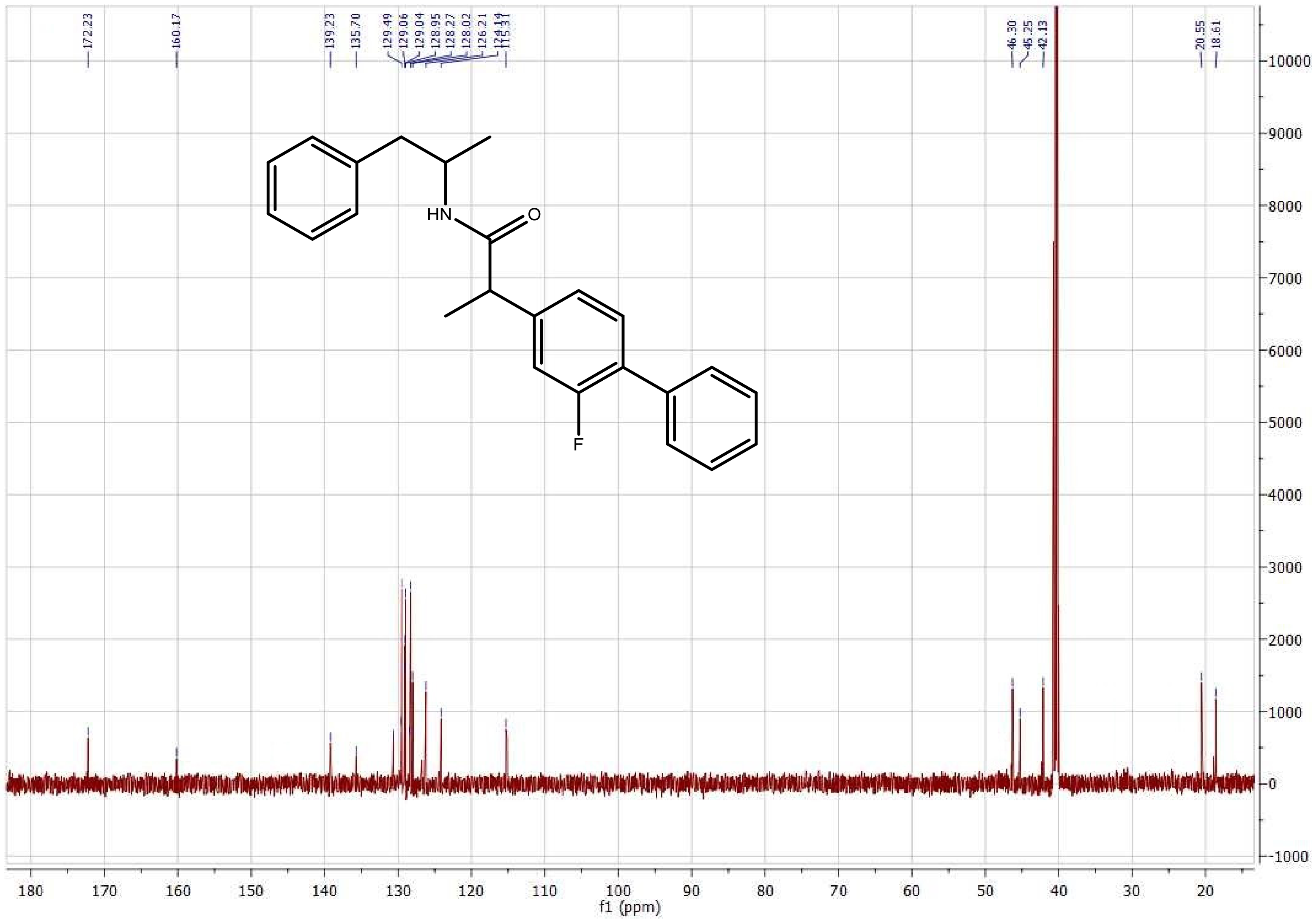

Figure S7. ^{13}C-NMR spectrum of compound **3b**.

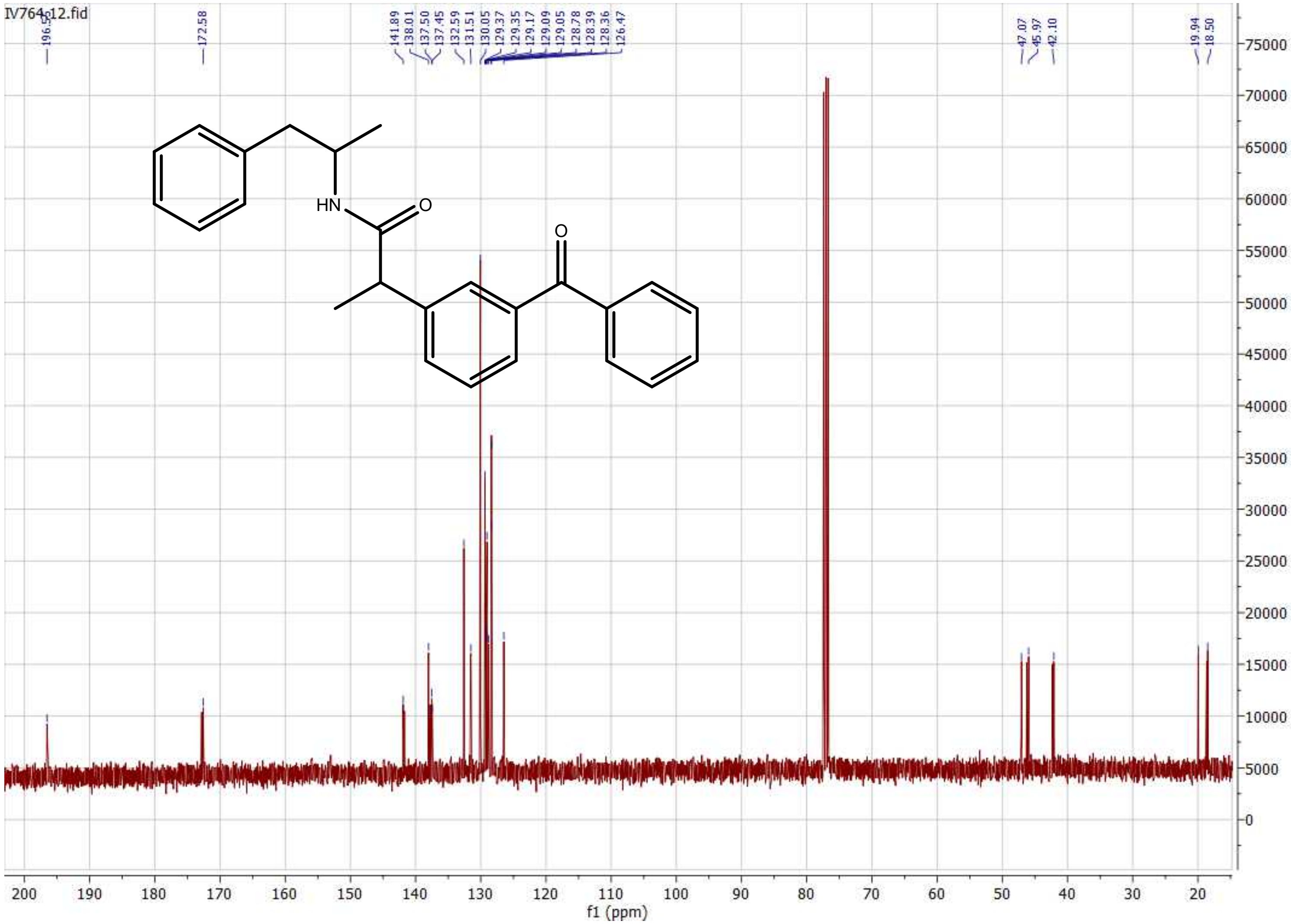

Figure S8. ^{13}C-NMR spectrum of compound **3c**.

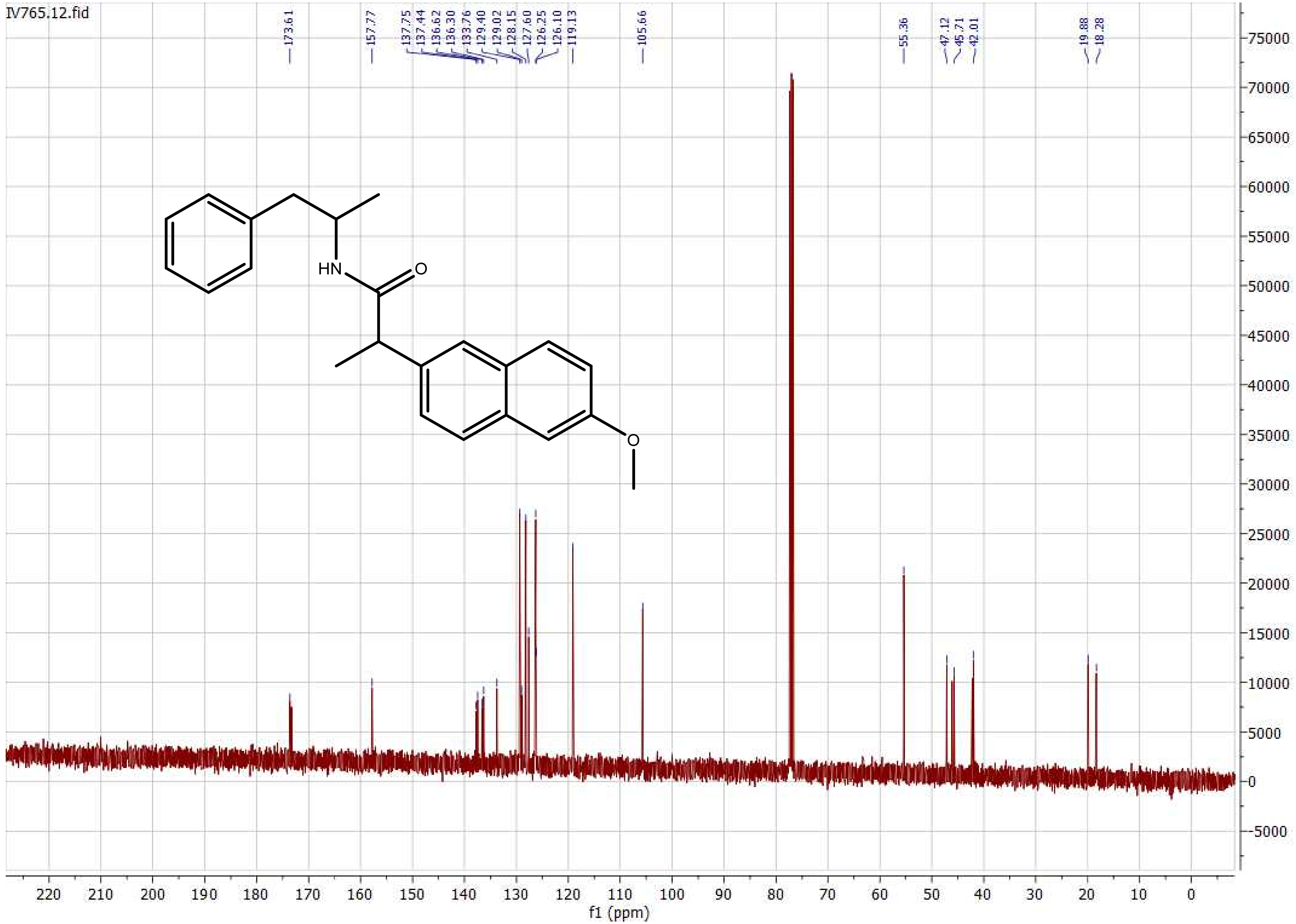

Figure S9. ^{13}C-NMR spectrum of compound **3d**.

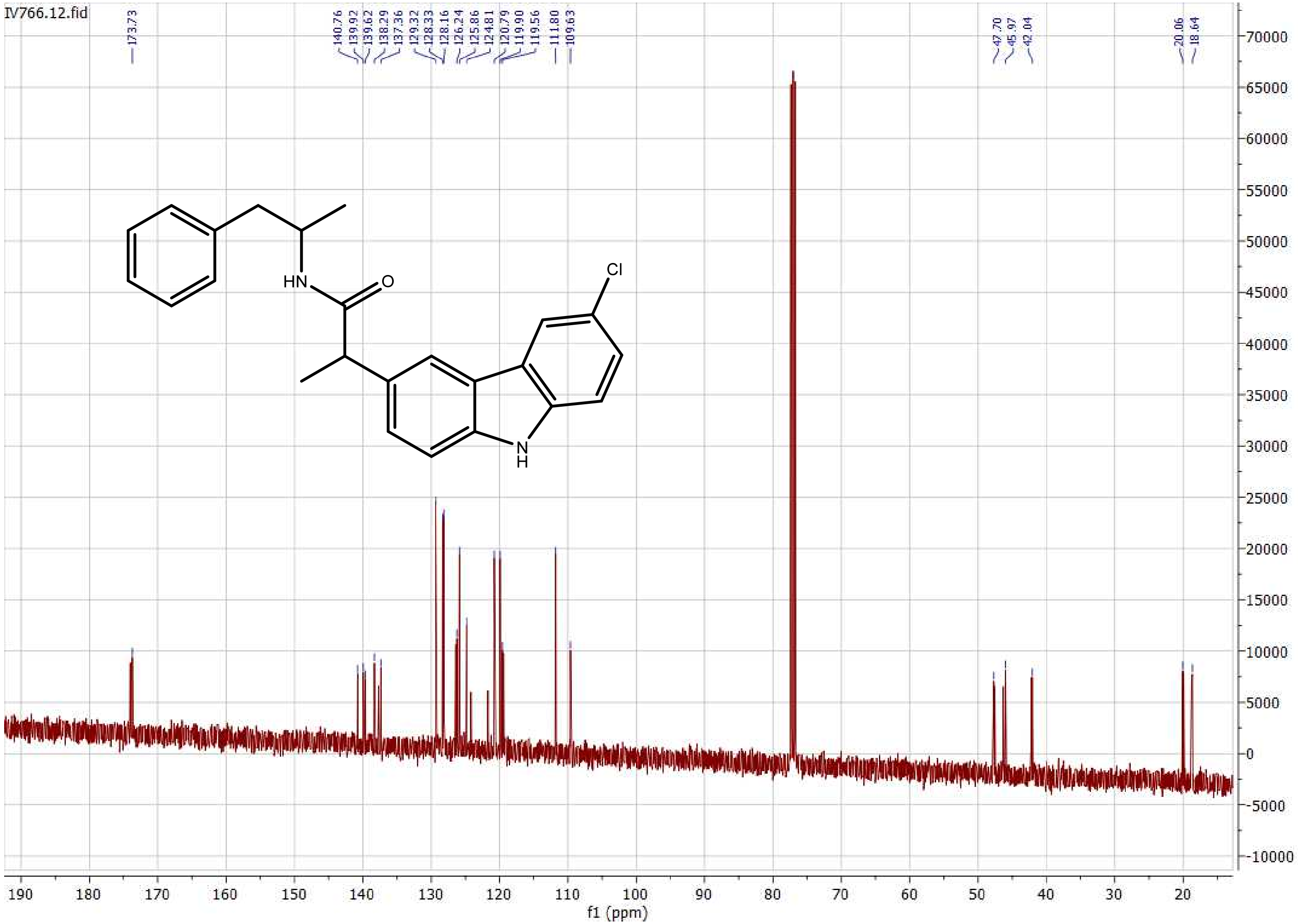

Figure S10. ^{13}C-NMR spectrum of compound **3e**.

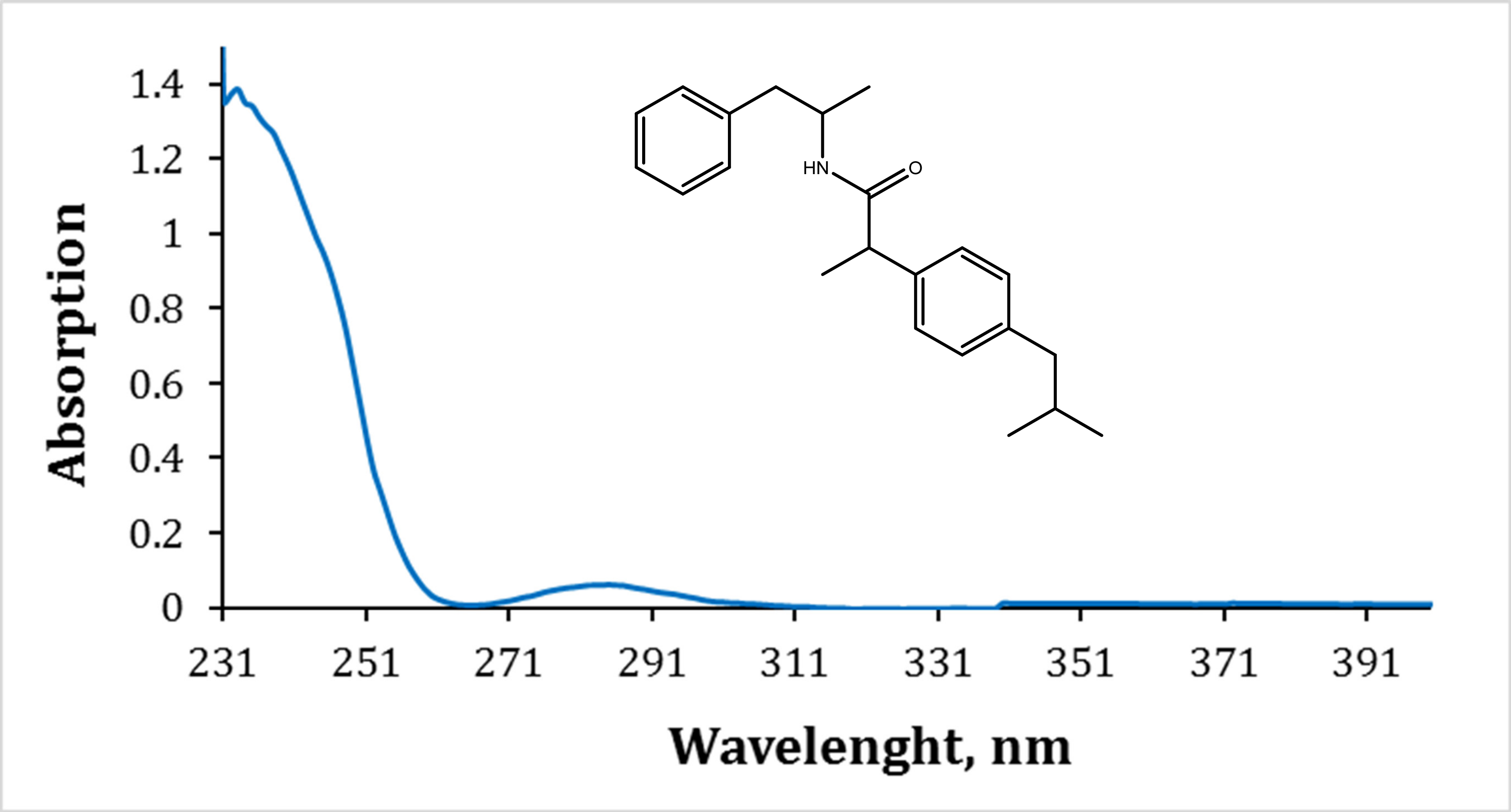

Figure S11. UV spectrum of compound **3a**.

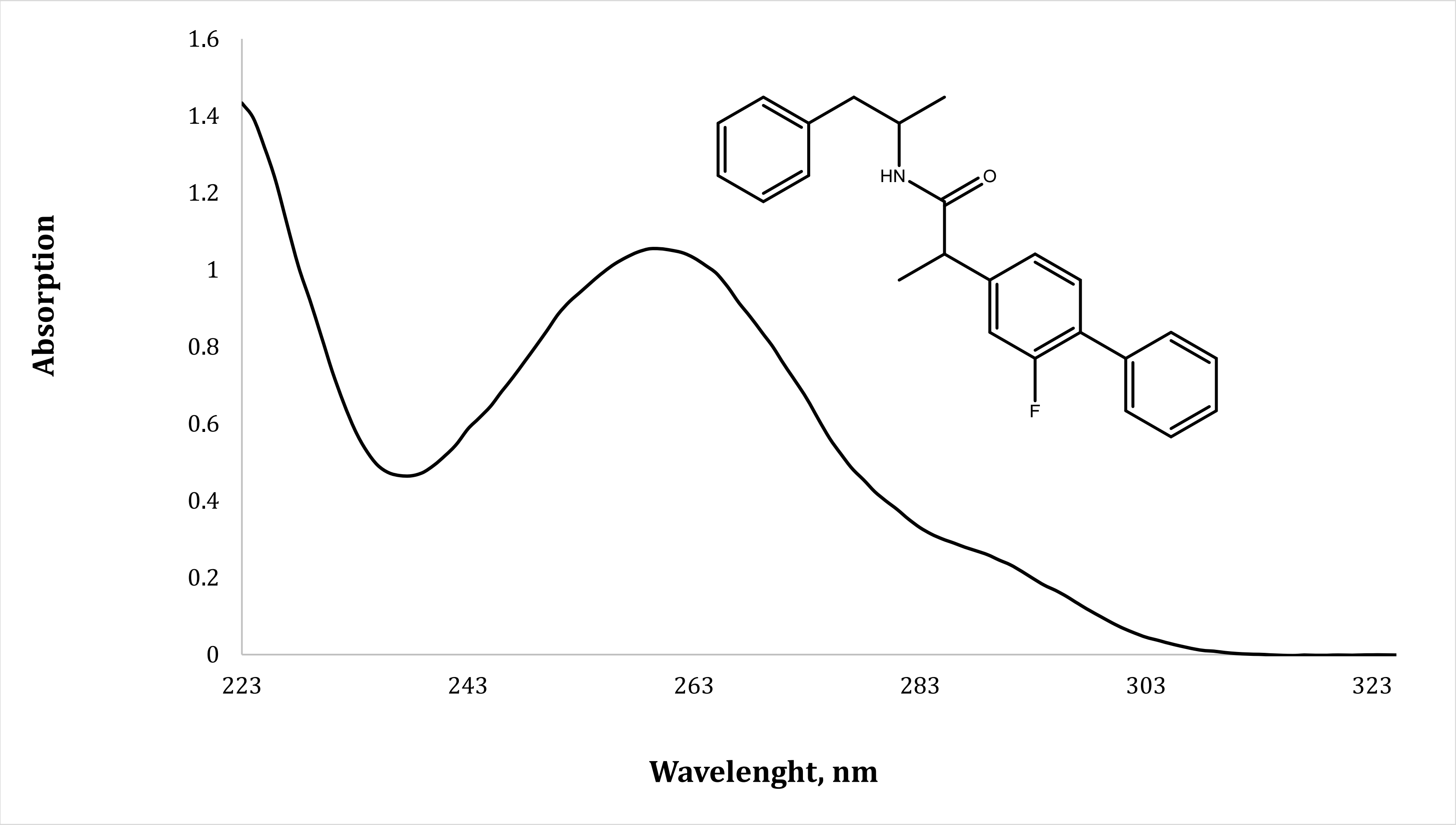

Figure S12. UV spectrum of compound **3b**.

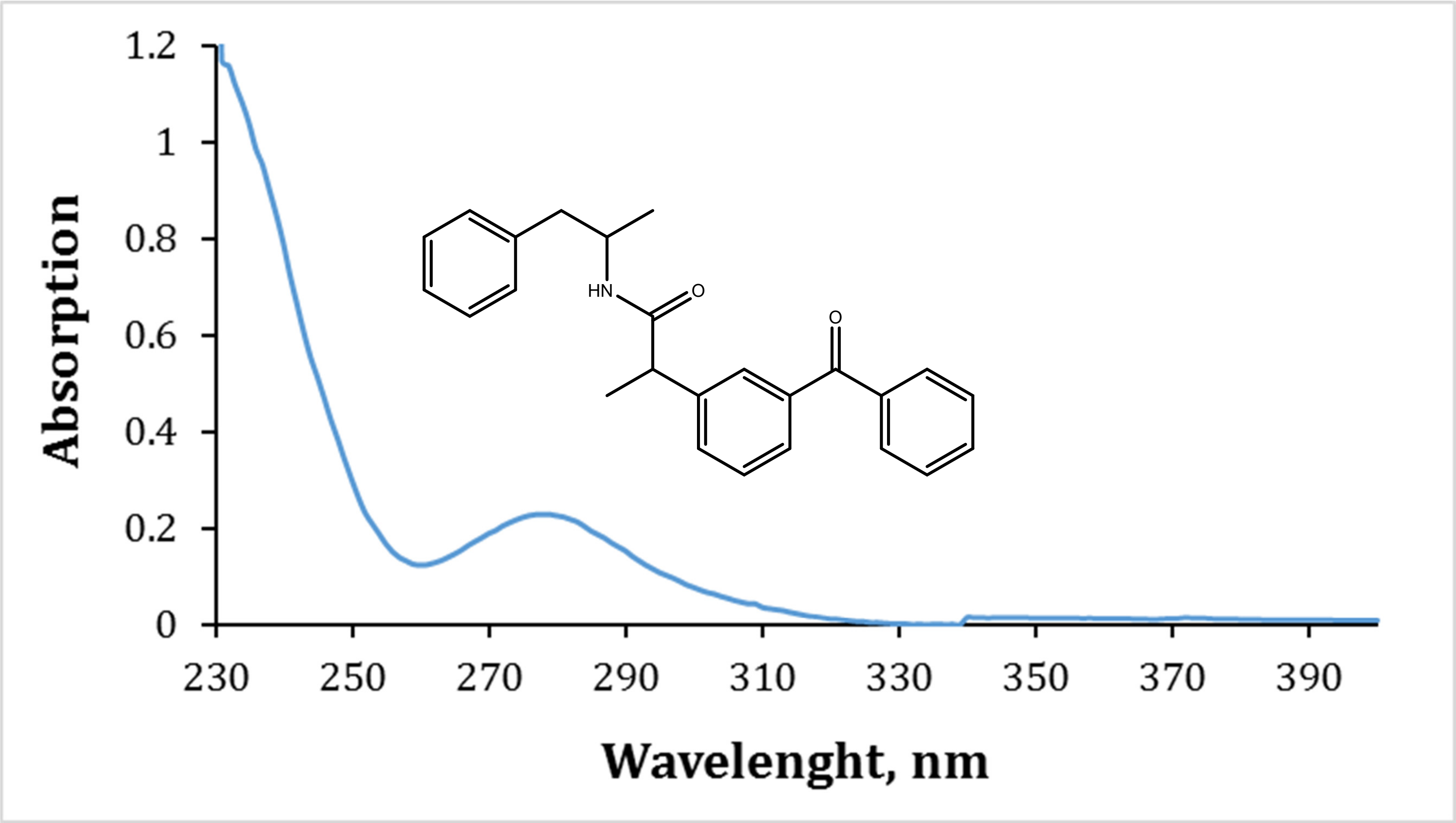

Figure S13. UV spectrum of compound **3c**.

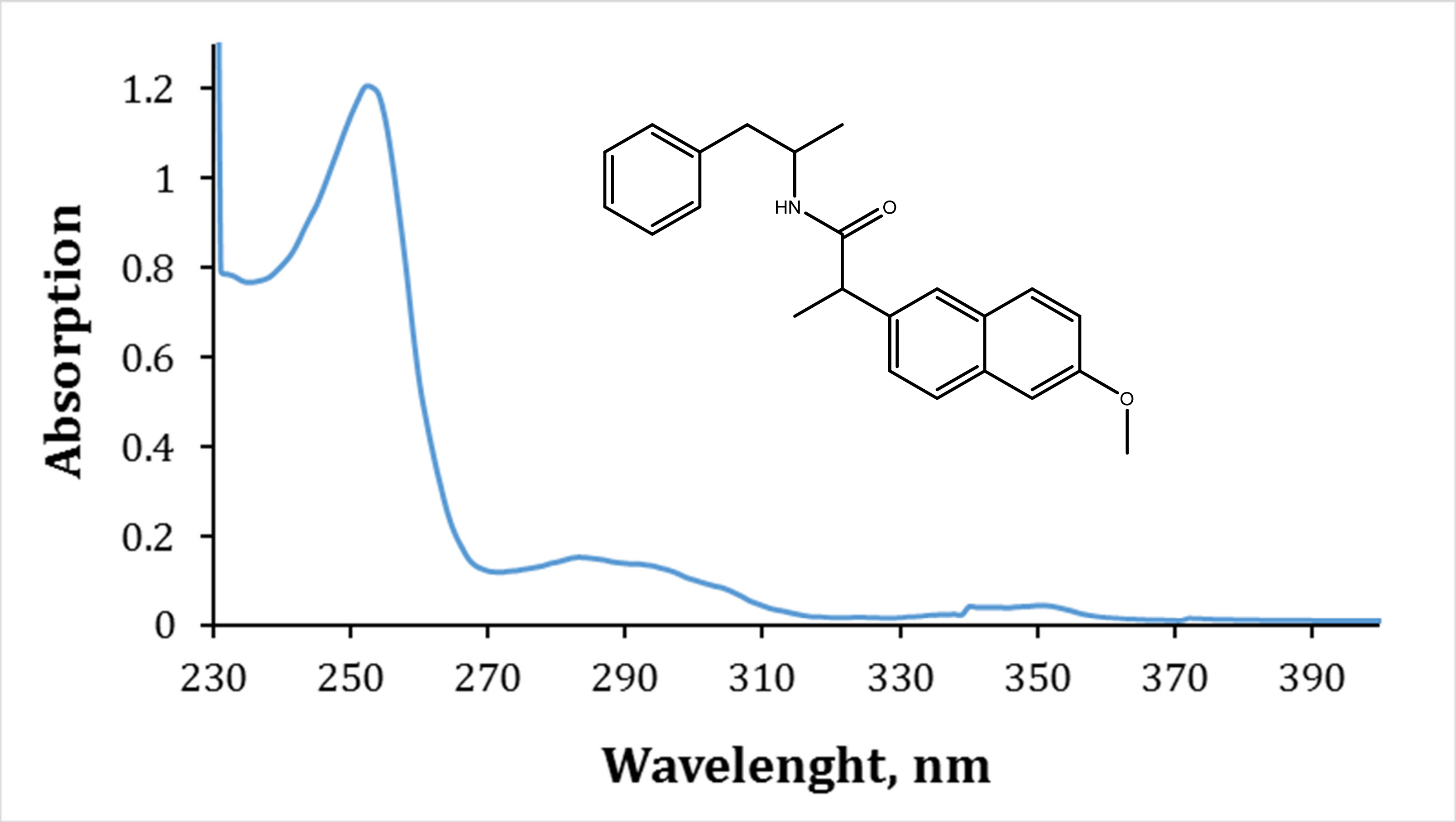

Figure S14. UV spectrum of compound **3d**.

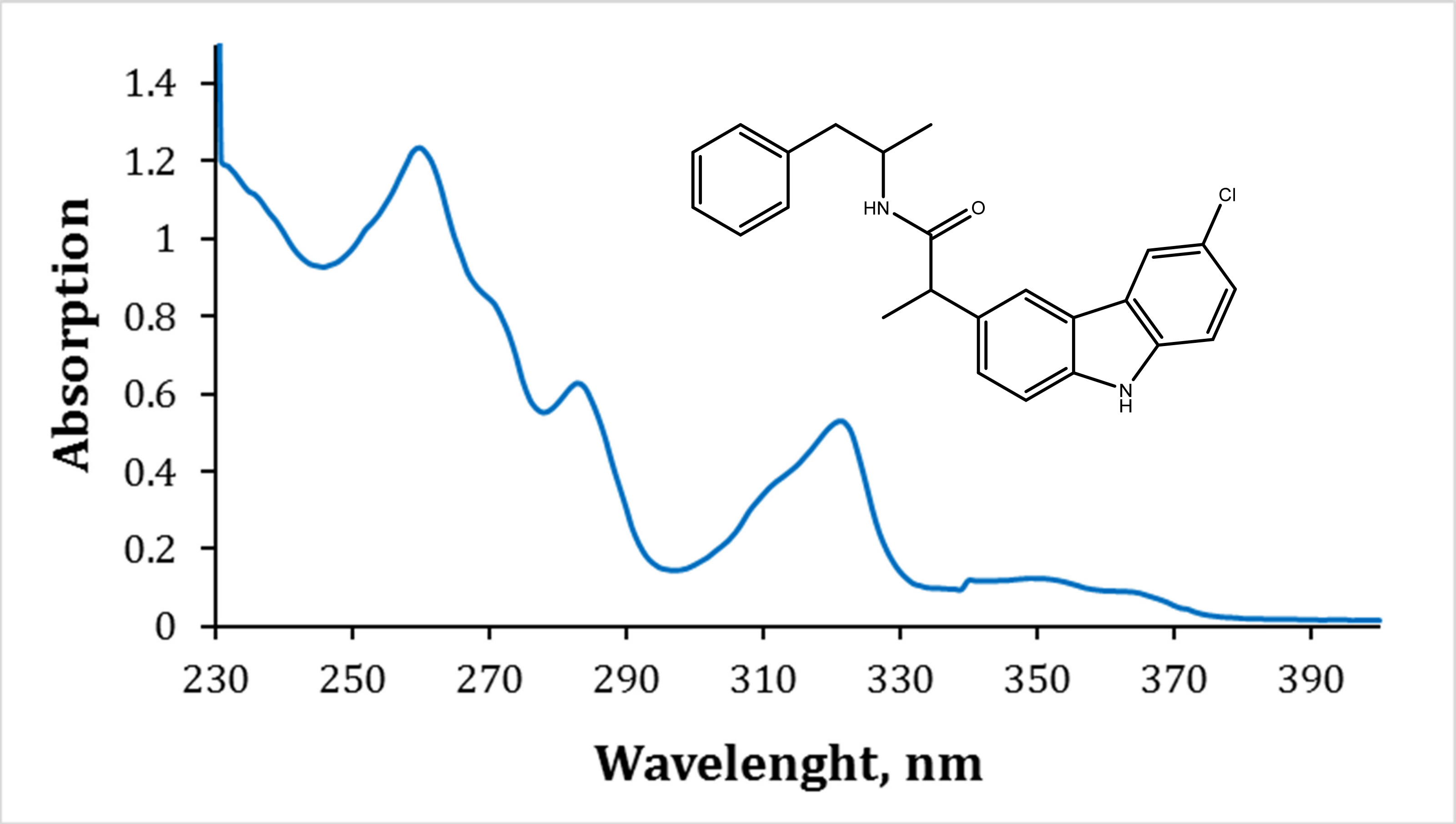

Figure S15. UV spectrum of compound **3e**.

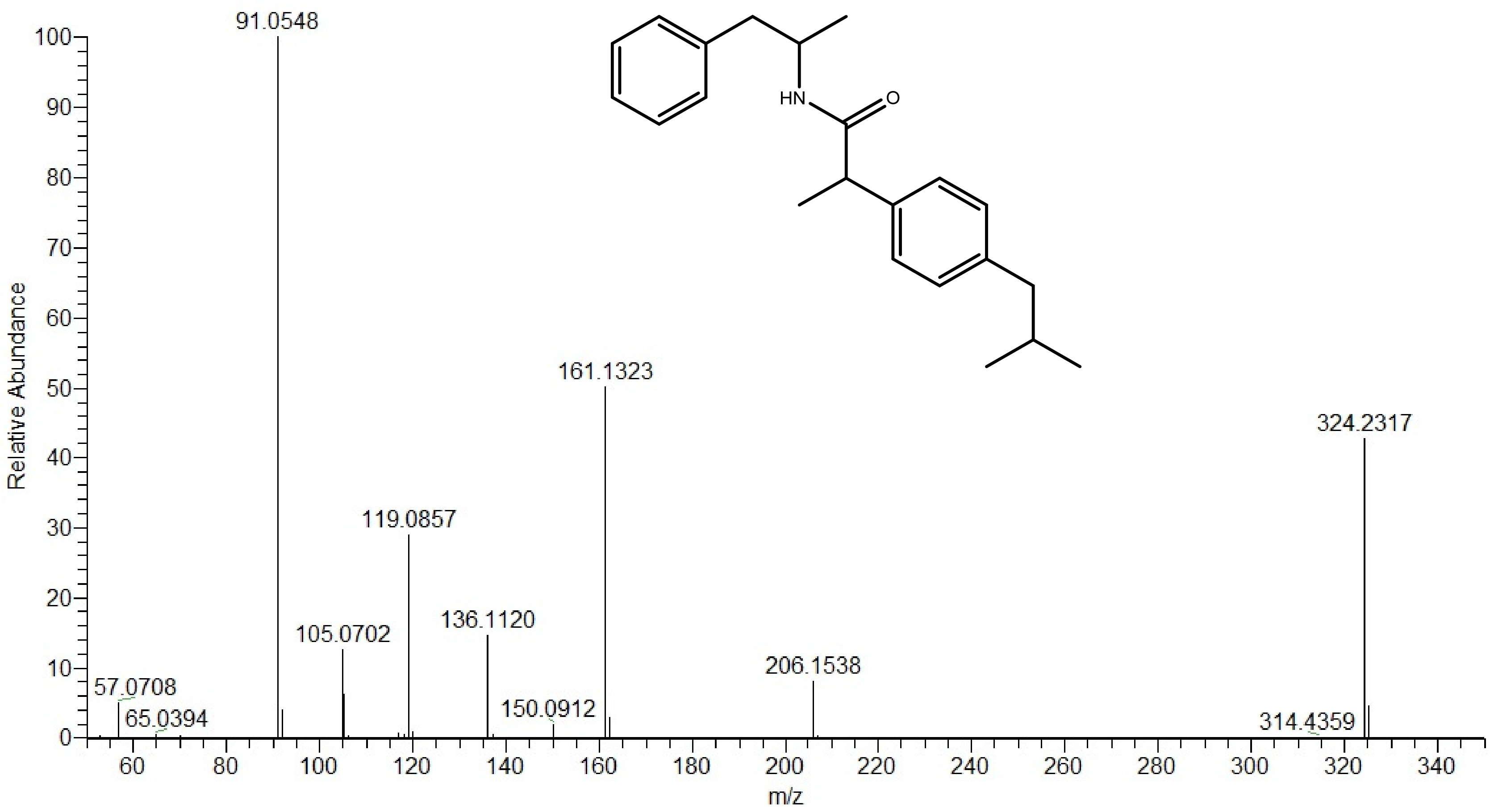

Figure S16. Mass spectrum of **3a** obtained by positive ion ESI-MS/MS.

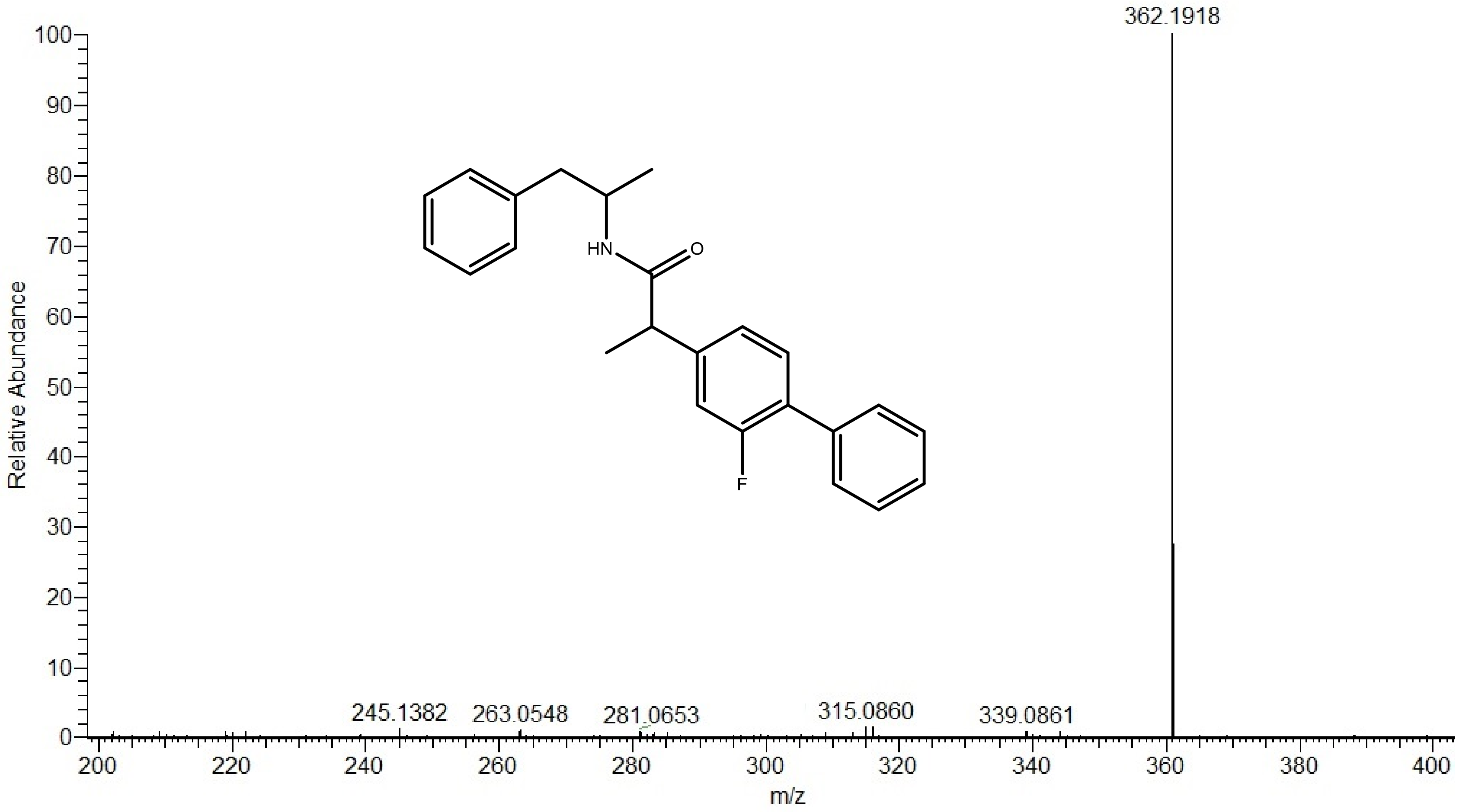

Figure S17. Mass spectrum of **3b** obtained by positive ion ESI-MS/MS.

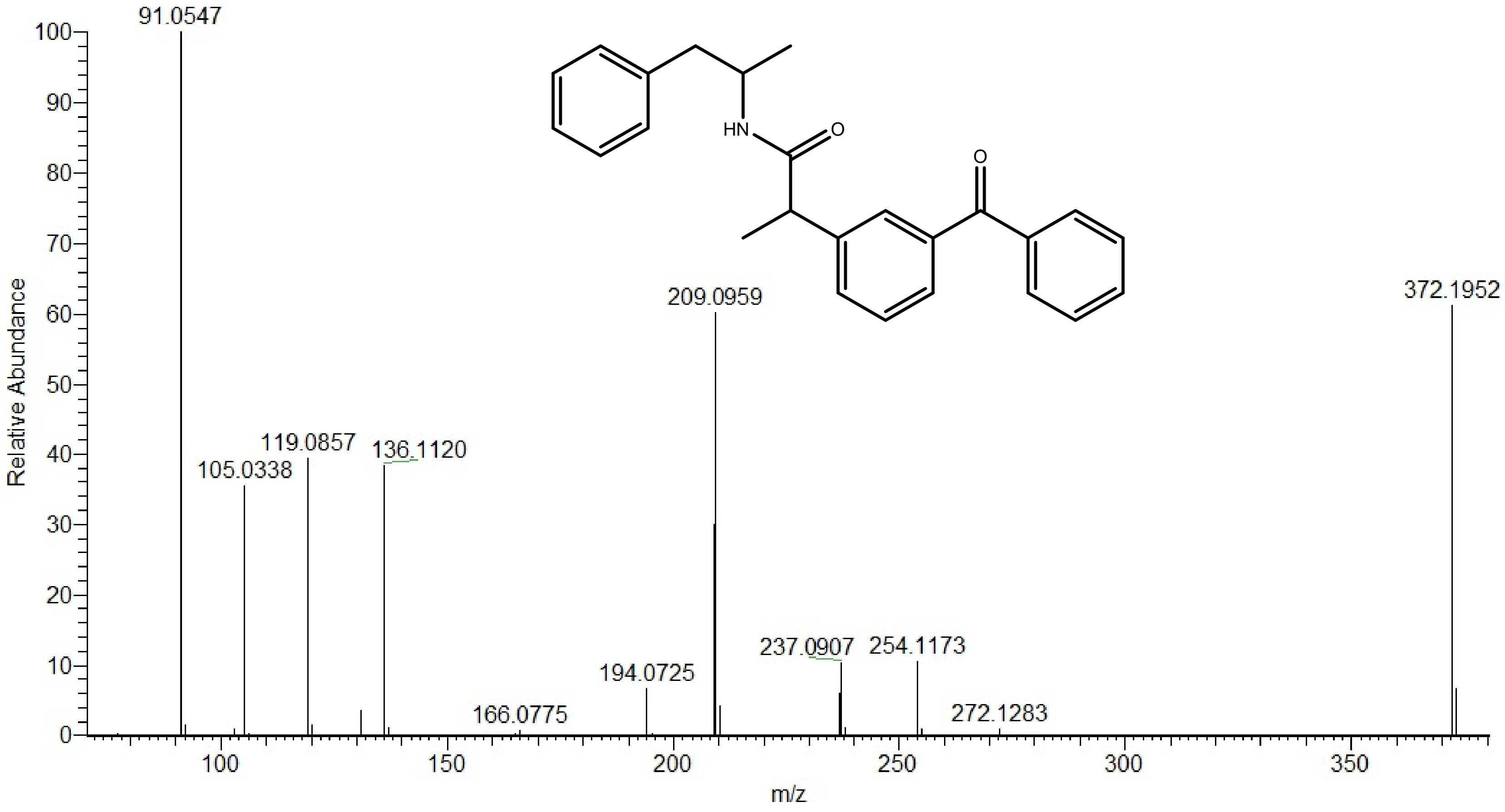

Figure S18. Mass spectrum of **3c** obtained by positive ion ESI-MS/MS.

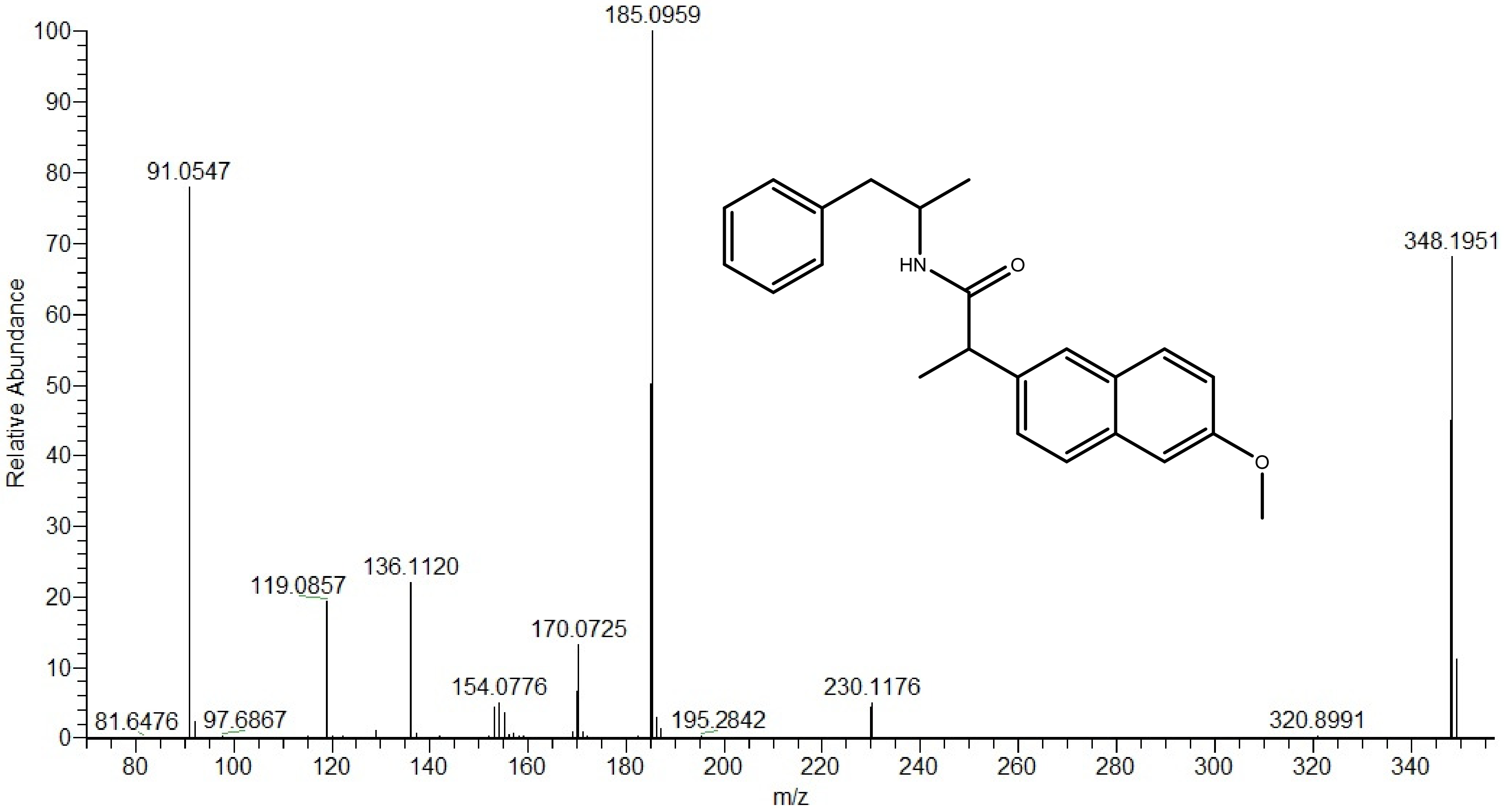

Figure S19. Mass spectrum of **3c** obtained by positive ion ESI-MS/MS.

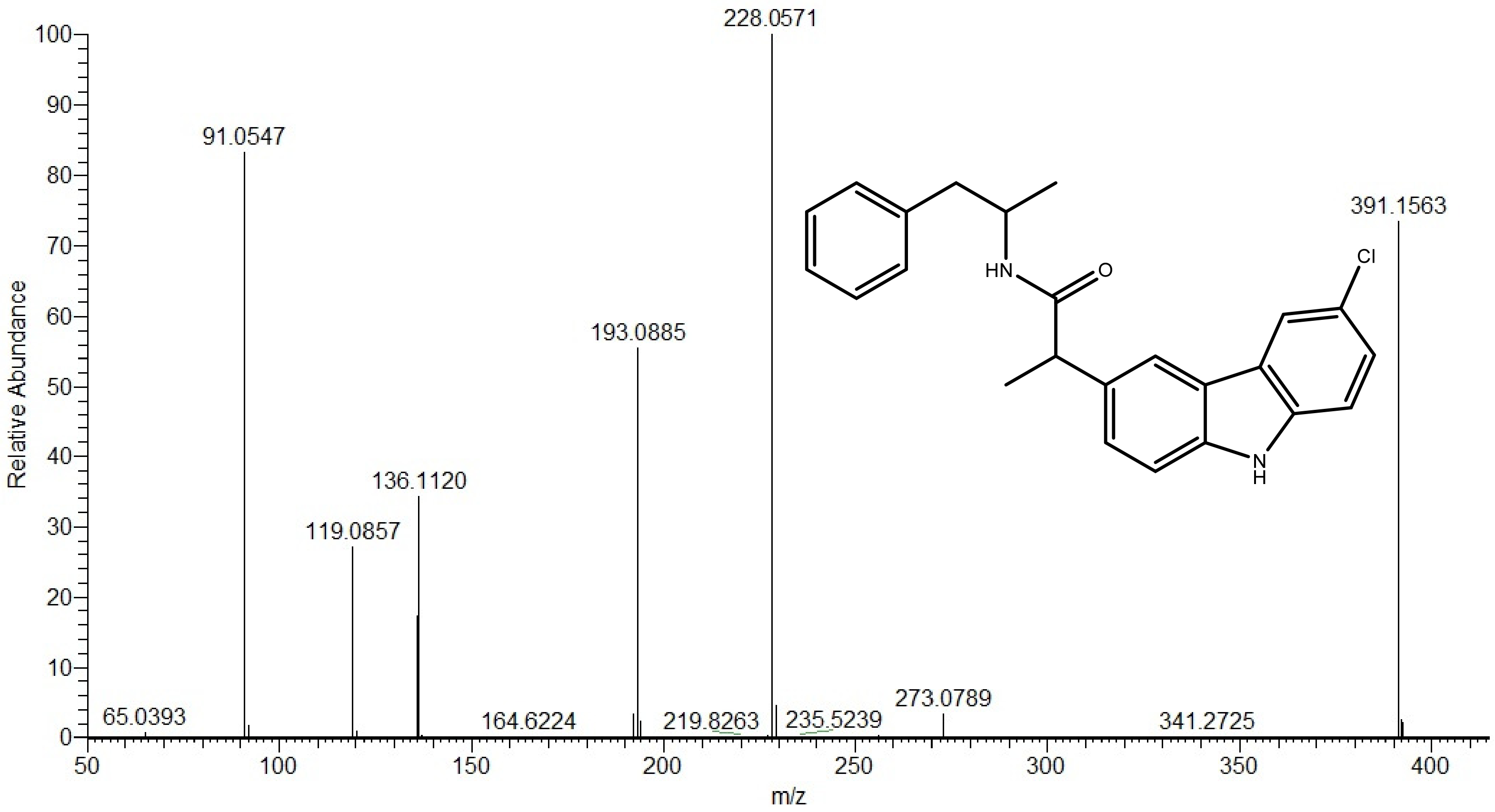

Figure S20. Mass spectrum of **3e** obtained by positive ion ESI-MS/MS.

References:

15. Manolov, S.; Ivanov, I.; Bojilov, D. (±) – 2 - (2-Fluoro-[1,1′-biphenyl]-4-yl) – N - (1-phenylpropan-2-yl) propanamide. *Molbank*, **2022**, 1, M1319. Doi: 10.3390/M1319.
